# Mitoferrin2 is a synthetic lethal target for chromosome 8p deleted cancers

**DOI:** 10.1186/s13073-024-01357-w

**Published:** 2024-06-17

**Authors:** Stephan Krieg, Thomas Rohde, Tobias Rausch, Luise Butthof, Lena Wendler-Link, Christoph Eckert, Kai Breuhahn, Bruno Galy, Jan Korbel, Maximilian Billmann, Marco Breinig, Darjus F. Tschaharganeh

**Affiliations:** 1 Helmholtz-University Group “Cell Plasticity and Epigenetic Remodeling”, German Cancer Research Center (DKFZ), Institute of Pathology, University Hospital Heidelberg, Heidelberg, Germany; 2 Institute of Human Genetics, University of Bonn, School of Medicine and University Hospital Bonn, Bonn, Germany; 3 European Molecular Biology Laboratory (EMBL), Genome Biology Unit, Heidelberg, Germany; 4 Institute of Pathology, University Hospital Heidelberg, Heidelberg, Germany; 5 Division of Virus-Associated Carcinogenesis, German Cancer Research Center (DKFZ), Heidelberg, Germany

**Keywords:** Synthetic lethality, Chromosome 8p deletion, SCNAs, MFRN1/2 paralog buffering

## Abstract

**Background:**

Somatic copy number alterations are a hallmark of cancer that offer unique opportunities for therapeutic exploitation. Here, we focused on the identification of specific vulnerabilities for tumors harboring chromosome 8p deletions.

**Methods:**

We developed and applied an integrative analysis of The Cancer Genome Atlas (TCGA), the Cancer Dependency Map (DepMap), and the Cancer Cell Line Encyclopedia to identify chromosome 8p-specific vulnerabilities. We employ orthogonal gene targeting strategies, both in vitro and in vivo, including short hairpin RNA-mediated gene knockdown and CRISPR/Cas9-mediated gene knockout to validate vulnerabilities.

**Results:**

We identified *SLC25A28* (also known as *MFRN2*), as a specific vulnerability for tumors harboring chromosome 8p deletions. We demonstrate that vulnerability towards MFRN2 loss is dictated by the expression of its paralog, *SLC25A37* (also known as *MFRN1*), which resides on chromosome 8p. In line with their function as mitochondrial iron transporters, MFRN1/2 paralog protein deficiency profoundly impaired mitochondrial respiration, induced global depletion of iron-sulfur cluster proteins, and resulted in DNA-damage and cell death. MFRN2 depletion in MFRN1-deficient tumors led to impaired growth and even tumor eradication in preclinical mouse xenograft experiments, highlighting its therapeutic potential.

**Conclusions:**

Our data reveal MFRN2 as a therapeutic target of chromosome 8p deleted cancers and nominate MFNR1 as the complimentary biomarker for MFRN2-directed therapies.

**Supplementary Information:**

The online version contains supplementary material available at 10.1186/s13073-024-01357-w.

## Background

Somatic genetic alterations are fundamentally involved in the development of cancer [1]. While gain and loss-of-function mutations resulting from single-base pair substitutions are widely acknowledged for their impact on tumorigenesis, cancer genomes are actually shaped in a much larger fraction by somatic copy-number alterations (SCNAs) [2, 3]. Among SCNAs, large megabase chromosomal deletions often lead to simultaneous loss of neighboring genes, some of which, i.e., tumor suppressors contribute to cancer progression, while others are mere bystanders [4]. Notably, recent findings have demonstrated the therapeutic potential of leveraging these bystander alterations through the concept of synthetic lethality (SL) [5]. A SL interaction between two genes occurs when perturbation of either gene alone does not alter cell fitness but concomitant perturbation of both genes induces a lethal phenotype. SL can be exploited as a powerful means to improve cancer therapy in that targeting one synthetic lethal partner may offer a favorable therapeutic window since healthy cells devoid of the associated functional alternation of one partner should not be impaired. A notable example of such an SL target gene pair is *PARP* and *BRCA1/2*, where *BRCA1/2* loss of function mutations sensitize tumor cells to pharmacological inhibition of *PARP* [6, 7]*.* Applying this concept to genes affected by SCNAs has been termed ‘collateral synthetic lethality’ [8]. Examples include *MTAP-PRMT5* interactions [9, 10], but remarkably also a large number of paralog pairs such as *ME2*-ME3, or *MAGOH-MAGOB* [11, 12]; a phenomenon termed paralog synthetic lethality: Given that paralogs oftentimes perform redundant cellular functions, one paralog can become indispensable if the function of the corresponding paralog is compromised [8].

By establishing a novel in silico analysis platform that integrates multiple publicly available large-scale genetics and functional genomics resources and by utilizing in vitro as well as in vivo genetic perturbation and epistasis experiments, we demonstrate that deletion of 8p, one of the most prevalent chromosomal deletions in solid cancers [4, 13], results in specific vulnerabilities towards loss of *MFRN2* (also known *SLC25A28*) function. MFRN2 is a mitochondrial iron transporter implicated in the transfer of iron into mitochondria, where it is used for essential anabolic processes such as iron-sulfur cluster (ISC) synthesis [14–16]. Interestingly, *MFRN2* has a functional paralog *MFRN1* (also known as *SLC25A37*), which resides on chromosome 8p and its expression is impaired as a result of chr8p deletions. Consequently, loss of both paralogs strongly impairs mitochondrial ISC-associated processes ultimately culminating in cell death, which can be exploited to eradicate MFRN1-deficient tumors. Thus, our results identify paralog synthetic lethality of MFRN1 and MFRN2 as the underlying mechanism of the striking dependency of chromosome 8p deleted cancers towards functional proficiency of MFRN2.

## Methods

### Cell culture

All cell lines were incubated at 37 °C with 5% CO_2_ and maintained in sterile conditions. Stock cells were cultured in Dulbecco’s modified Eagle’s medium (DMEM) containing 4500 mg/L glucose, L-glutamine, sodium pyruvate, and sodium bicarbonate supplemented with 10% FCS (Sigma-Aldrich) and 1% penicillin/streptomycin (10,000 U/ml penicillin and 10 mg/ml streptomycin, Sigma-Aldrich). For all experiments involving inducible TRE-dependent shRNAs, cells were cultivated in complete DMEM supplemented with 1 µg/mL doxycycline (DOX) that was changed every 48 h. For time course experiments involving DOX inducible constructs, cells were cultured in DOX-containing medium for 36 h prior to time course start for up to 12 days.

### Primary cell culture

Primary murine liver tumor cell lines were isolated from sterile resected and digested liver tumors as previously described [17]. In brief, liver tumors were resected with sterile instruments and washed in sterile PBS prior to digestion. Then tumor tissue was minced and resuspended in a mix of 4 mg/mL collagenase and dispase (w/v in sterile, serum-free Dulbecco’s Modified Eagle’s medium (DMEM, Sigma)) at 37 °C for 30 min with gentle shaking. The dissociated tumor cells were then washed with complete DMEM (supplemented with 10% (v/v) fetal bovine serum and 1% penicillin/streptomycin) and plated on collagen-coated plates (PurCol, Cell Systems; 0.05 mg/mL). Primary cultures were passaged until visibly free from other contaminating cell types.

### Molecular cloning

Lentiviral overexpression construct for Mfrn1 was generated using NEBuilder® HiFi DNA Assembly of a PCR product generated from (pENTR223-SLC25A37) obtained from DKFZ core facility and (pDEST6.2/V5-DEST).

Guide cloning and shRNA cloning were performed as previously described [17]. Briefly, individual single guide RNAs (sgRNA) for CRISPR/Cas9 effectors were designed using the web tool CHOPCHOP [18] and subcloned into the respective vectors.

For effective RNAi-mediated interference of target genes, potent shRNAs were predicted using the SplashRNA algorithm described by Pelossof et al. [19] and ligated into the respective backbones. PCR amplified and digested oligomers were ligated into the MlPe construct to ensure constitutive hairpin expression.

To test sgRNA and shRNA potency, target cells were transduced with a low MOI (< 0.7) to allow single integration and selected with Puromycin. Knockdown efficiency was analyzed by immunoblotting and qRT-PCR, comparing cells expressing targeted shRNA to Renilla luciferase-expressing cells. The two most potent shRNAs were cloned into DOX-inducible constructs (LT3-GEPIR) and used for further downstream analyses. To validate correct and effective site-specific cleavage of DNA strands by the CRISPR/Cas9 sgRNAs the T7 endonuclease1 mismatch detection assay was performed as previously described [17].

### Virus production and transduction

Virus production, transduction, and selection were performed as previously described [17].

### gDNA isolation

Isolation of genomic DNA was performed using the Puregene® Core Kit A (Qiagen) according to the manufacturer’s protocol.

### Immunoblotting

Immunoblotting was performed as previously described [17].

The following antibodies were used:

**Table:** 

Target	Species	Manufacturer	cat.#	Dilution
Primary antibodies used for immunoblotting				
ACTIN	HRP-conjugated	Sigma-Aldrich	A3854	1:20,000
ACO2	Rabbit	Cell Signaling Technologies	cs6922	1:1000
ALAS1	Rabbit	Proteintech	16,200–1-AP	1:1000
Cleaved Caspase 3	Rabbit	Cell Signaling Technologies	cs9661	1:1000
Cleaved PARP	Rabbit	Cell Signaling Technologies	cs5625	1:1000
DMT1	Rabbit	Proteintech	26,312–1-AP	1:500
FECH	Mouse	Proteintech	14,466–1-AP	1:1000
FTH1	Rabbit	Cell Signaling Technologies	cs3998	1:1000
FXN	Rabbit	Abcam	ab175402	1:1000
GFP	Rabbit	Cell Signaling Technologies	cs2555	1:1000
IRP1	Rabbit	Cell Signaling Technologies	cs20272	1:1000
IRP2	Rabbit	Cell Signaling Technologies	cs37135	1:1000
ISCU	Rabbit	Proteintech	14,812–1-AP	1:1000
MFRN1	Rabbit	Proteintech	26,469–1-AP	1:1000
MFRN2	Rabbit	Abcam	ab277516 **Discontinued!**	1:500
OXPHOS	Mouse	Abcam	ab110411	1:500
TRANSFERRIN	Rabbit	Proteintech	10,084–2-AP	1:1000
VDAC	Rabbit	Cell Signaling Technologies	cs4661	1:1000
VINCULIN	Mouse	Sigma-Aldrich	V9131	1:4000
γH2AX	Mouse	Merck Millipore	05–636	1:200
Secondary antibodies used for immunoblotting				
Goat polyclonal anti-mouse secondary antibody	HRP-conjugated	Jackson Immuno Research, 115–035-008	1:5000	
Goat polyclonal anti-rabbit secondary antibody	HRP-conjugated	Jackson Immuno Research, 115–035-003	1:5000	

### Immunofluorescence (IF)

Intracellular staining of yH2AX foci was analyzed by indirect IF and fluorescence microscopy. SNU387 cells were seeded in a density of 1 × 10^5^–5 × 10^5^ cells in 6-well plates on coverslips. For fixation, the culture medium was aspirated and cells were washed twice in 1 × PBS before adding 4% PFA in 1 × PBS for 5–8 min depending on the cell line. After fixation, cells were washed twice in 1 × PBS and permeabilized in 0.5% Triton X-100 in 1 × PBS for no longer than 5 min. Permeabilization was followed by a two-time wash in 1 × PBS and subsequent blocking in either 5% BSA or 5% goat serum in 1 × PBS supplemented with 0.3 M of Glycine and 0.1% Triton X-100 for 20 min. In the meantime, primary antibodies of different host species were diluted as indicated below in the blocking buffer. After blocking, cells were taken out of the culture dish and transferred to parafilm with cells facing up before adding 50 µl of respective primary antibody dilution to the coverslips. The cells were incubated for 1 h at RT followed by a four-time wash in 1 × PBS. The respective secondary antibodies conjugated with Alexa Fluor 594 were diluted 1:450 in 1 × PBS and mixed with a 1:20,000 dilution (1 µg/mL) of a 20-mM stock solution of Hoechst. The coverslips were incubated with 50 µl of secondary antibody mixture for 45 min at RT in the dark. The cells were then washed five times in 1 × PBS and mounted on a microscope slide cell facing down with 15 µl Mowiol® (Calbiochem). To let the Mowiol® harden, the microscope slides were kept at RT for 30 h until analysis. For long time storage, coverslips were sealed with nail polish and kept at 4 °C. Fluorescence was analyzed using the AXIO Observer.Z1 Fluorescence Phase Contrast Microscope (Zeiss, Germany) and processed with the ImageJ software.

**Table:** 

Target	Species	Manufacturer	cat.#	Dilution
Antibodies used for indirect immunofluorescence				
γH2AX	mouse	Merck Millipore	05–636	1:200
Hoechst		Sigma-Aldrich	33,342	1:20,000
Alexa Fluor® 594-conjugated a-mouse	goat	Invitrogen	A-11032	1:450

### Quantitative reverse transcription PCR (qRT-PCR)

qRT-PCR was performed as previously described [17]. Following primers were used: Mfrn1_F: TTGAATCCAGATCCCAAAGC;

Mfrn1_R: GTTTCCTTGGTGGCTGAAAA;

Mfrn2_f: TCGTCAAGCAGAGGATGCAGAT; and.

Mfrn2_R: GTTAAAGTGCTCTTGCAGGAAC.

### Colony formation assay

CFA was performed as previously described [17]. The initial cell number for seeding of each cell line in different experiments is provided in respective figure legends.

### Competition assay

Competition assays were performed as previously described^**17**^. Briefly, human and murine SpCas9 competent cell lines expressing either a non-targeting sgRNA (sgCTR) or a sgRNA targeting MFRN1 were mixed with lentiviral constructs expressing either a non-targeting sgRNA or a sgRNA targeting MFRN2 together with GFP in a ratio of 30:70. For knockdown-dependent competition assays, the Cas9-dependent single-KO cells of MFRN1 or sgCTR cells were mixed in the same ratio with cells expressing either a shRNA targeting Renilla luciferase (shRen) or MFRN2 together with GFP in a DOX-dependent manner. The stimulation time with 1 µg/mL DOX began 36 h prior to the assay start and the DOX-containing medium was changed every 2 days. The cells were seeded in a final concentration of 2 × 10^5^ cells in 6-well plates with 3 technical and 3 biological replicates. The acquisition and analysis were performed using the guava easyCyte™ HT system.

### Mitochondrial-cytosolic fractionation

Abcam’s Mitochondrial/ Cytosolic Fractionation Kit (ab65320) and the Mitochondria Isolation Kit (Thermo Scientific, cat.:89,874) were used according to the manufacture’s protocol. In total 6 × 10^7^ cells were processed for each individual sample using pellet pestles (Sigma-Aldrich, cat.: Z359971) for dounce homogenization in 1.5 mL reaction tubes and proceeding with downstream analysis.

### Apoptosis assay using Caspase-Glo® 3/7

To analyze Caspase cleavage after a genetic alteration of cultured cells, Caspase-Glo® 3/7 Assay (Promega, G8091) was used according to the manufacturer’s protocol. Briefly, after defined timepoints during DOX treatment triplicates of 1.5 × 10^4^ SNU387 or 2 × 10^4^ PLC cells were seeded in 100 µl into opaque 96-well microplates (OptiPlate™, PerkinElmer). At the day of the measurement, 100 µl of Caspase-Glo® 3/7 luminescence substrate was added to each well incubated for 1 h at RT and analyzed using the EnSpire® Multimode Plate Reader (PerkinElmer).

### Cell cycle analysis

To analyze changes in the cell cycle distribution after loss of MFRN1 and MFRN2, cellular DNA content was measured near 500 nm emission using a flow cytometer and 4′,6-diamidino-2-phenylindole (DAPI) as a DNA-intercalating agent. At a specific timepoint, after DOX treatment cells were harvested by collecting the culture medium together with the trypsinized cells, washed using 1 × PBS, and transferred into 1.5-ml reaction tubes. Cells were resuspended in 100 µl ice-cold PBS and 900 µl ice-cold 80% EtOH added in a dropwise fashion while vortexing to allow fixation. Cells were stored at − 20 °C for at least 24 h before further processing. At the day of measurement, EtOH fixed cells were centrifuged down, rehydrated in 1 ml PBS for 15 min, and cell number adjusted to 5 × 10^5^ cells. For DNA staining, cells were resuspended in 300 µl PBS-containing 1 µg/ml DAPI and 0.1% (v/v) TritonX-100. After 30 min incubation at RT in the dark, the cell suspension was filtered through a cell strainer cap (Falcon, cat:352,235) and analyzed at the BD LSR Fortessa flow cytometer (BD, Germany) using the BD FACS Diva software v8.0.1. Analysis and image generation were performed with FlowJo v10.

### SA-ß-gal Senescence assay

Cytochemical detection of ß-galactosidase (ß-gal) activity in SNU387 and PLC cells was accomplished by using the chromogenic substrate X-gal and measuring its conversion to a blue indigo dye upon ß-gal cleavage at sub-physiological pH-conditions (pH = 6) [20]. For ß-gal detection, cells were seeded in the appropriate density in complete DMEM in triplicates and 6-well plates 1 day prior to treatment. After treatment, cells were incubated for at least 10 days before staining and subsequent fixation. After incubation, cells were washed twice in 1 × PBS to remove all residual medium. Then the fixation solution containing 2% (v/v) formaldehyde and 0.2% (v/v) glutaraldehyde was added and incubated for no longer than 5 min to avoid destruction of ß-gal activity. The fixed cells were washed twice with 1 × PBS and once in dH_2_O followed by incubation in staining solution (for 12–16 h without CO_2_ at 37 °C. After incubation, the staining solution was aspirated and cells were washed twice in 1 × PBS followed by a one-time wash in MeOH. Stained and fixed cells were allowed to air dry and subsequently analyzed by bright-field microscopy using the AXIO Observer.Z1 Fluorescence Phase Contrast Microscope (Zeiss, Germany).

### Aconitase assay

Aconitase activity was measured spectrophotometrically using the Aconitase assay kit (Abcam, ab109712). Utilizing the increase in absorbance at 240 nm associated with the formation of the cis-aconitate, the aconitase activity was inferred.

Inducible hairpin harboring cells were grown in a DOX-containing medium for 6 days in triplicates. On the day of the assay, 1 × 10^6^ cells were harvested, washed with ice-cold PBS, and resuspended in 100 µl aconitase preservation solution supplemented with detergent for lysis. After 30 min incubation on ice, cells were centrifuged at 4 °C and 20,000 g for 10 min. To allow input of equal amounts of protein, protein concentration was determined as described in Sect. 3.1.3.2. Fifty microliters of the individual equilibrated lysates were transferred into the wells of the assay plate, and 200 µl of assay buffer was added and measured on a plate reader (SPECTROStar Nano microplate reader, BMG Labtech) using a kinetic program.

### Calcein assay

To determine changes in intracellular iron levels, cells were incubated with Calcein-AM (Thermo Scientific, C1429) a cell membrane permeable esterase substrate, and turnoff fluorescent probe of iron ions. For that, cells were cultured in a DOX-containing medium for 6 days. One day prior to measurement, cells were split and either treated with 100 µM CPX (Sigma-Aldrich) and 100 µM DFO (Sigma-Aldrich) or left untreated. On the day of the measurement, cells were harvested and resuspended in completed DMEM-containing 1.5 µM of Calcein-AM and incubated for 45 min at 37 °C. The cell suspension was filtered through a cell strainer cap (Falcon, cat: 352,235) and analyzed at the BD LSR Fortessa flow cytometer (BD, Germany) using the BD FACS Diva software v8.0.1. Analysis and image generation were performed with FlowJo v10. To determine the difference in the labile iron pool MFI of CPX/DFO treated samples was subtracted from the non-treated samples.

### Measurement of mitochondrial respiration

Measurement of the effect of MFRN1-MFRN2 loss on mitochondrial respiration was accomplished using the Seahorse XF96 Cell Mito Stress Test and the Seahorse extracellular flux analyzer (Agilent Technologies) [21]. Briefly, 1 day prior to assay measurement and time course completion doxycycline-treated cells were seeded in a volume of 100 µl per well into the Sea horse XF96 Cluture Microplate to allow cells to reach 80–90% confluency. Simultaneously, the sensory cartridge of the Extracellular Flux assay kit was hydrated using 200-µl Seahorse XF Calibrant per well and incubated at 37 °C in non-CO_2_ conditions for 12–18 h. On the day of the measurement, 100 mL of Sea horse assay medium was prepared by adding 1 mL of 1 g/L glucose, 1 mL 100 mM pyruvate solution, and 1 mL 200 mM glutamine solution followed by a 3-time wash of the culture microplate with 180 µl assay medium. To de-gas the cultured cells, the culture microplate was incubated in non-CO_2_ conditions 1 h prior to measurement. During the last 20 min before measurement, XF Cell Stress Compounds were prepared and loaded in the hydrated cartridge. For that, 20 µl of 2 µM Oligomycin was loaded into port A, 22 µL of 1 µM FCCP was loaded into port B and 25 µl of 0.5 µM Rotenenone/antimycin A was loaded into port C. Using the Seahorse XFe96 extracellular flux analyzer (Agilent Technologies, USA) and pre-installed Mito Stress Test template, the oxygen consumption rate (OCR) as well as the extracellular acidification rate (ECAR) was measured. The assay data was analyzed using the Seahorse Wave software.

### Mass spectrometry-based protein analysis

To analyze changes in the proteome after experimental manipulation of cultured cells, whole-cell lysates were submitted to mass spectrometry-based protein analysis. Protein extraction was performed by directly adding a complete RIPA-lysis buffer solution to the cells, collecting the primary lysates, and proceeding to determine the protein concertation. Sample digestion, measurement, and statistical analysis were performed by the proteomics core facility (Mass Spectrometry based Protein Analysis Unit) of the DKFZ Heidelberg. Briefly, sample preparation for MS-based proteome analysis was performed using In-gel digestion. In the first step, the protein samples were run into an SDS-PAGE-gel for 0.5 cm. After Coomassie staining, the total unfractionated sample was cut out and used for subsequent Trypsin digestion according to a modified protocol described by Shevchenko et al. on a DigestPro MSi robotic system (INTAVIS Bioanalytical Instruments AG) [22]. Peptides were re-suspended in a loading buffer containing 2.5% 1,1,1,3,3,3-Hexafluoro-2-propanol, 0.1% TFA in water. LC–MS/MS analysis was carried out on an Ultimate 3000 UPLC system (Thermo Fisher Scientific) directly connected to an Orbitrap Exploris 480 mass spectrometer. Peptides were online desalted on a trapping cartridge (Acclaim PepMap300 C18, 5 µm, 300 Å wide pore; Thermo Fisher Scientific) for 3 min using 30 µl/min flow of 0.05% TFA in water. The analytical multistep gradient was carried out on a nanoEase MZ Peptide analytical column (300 Å, 1.7 µm, 75 µm × 200 mm, Waters) using solvent A (0.1% formic acid in water) and solvent B (0.1% formic acid, 80% acetonitrile in water). For 134 min, the concentration of B was linearly ramped from 2 to 38%; followed by a quick ramp to 95%; then after 2 min, the concentration of B was lowered to 2%; and a 10-min equilibration step was appended. The eluting peptides were analyzed in the mass spectrometer using the data-depend acquisition (DDA) mode. A full scan at 60 k resolution (380–1400 m/z, 300% AGC target, 45 ms maxIT) was followed by up to 1 s of MS/MS scans. Peptide features were isolated with a window of 1.4 m/z, fragmented using 26% Normalized Collision Energy (NCE). Fragment spectra were recorded at 15 k resolution (100% AGC target, 22 ms maxIT). Unassigned and singly charged eluting features were excluded from fragmentation and dynamic exclusion was set to 35 s.

Data analysis was performed as described by Ganing et al [23]. Briefly, using an organism-specific database extracted from Uniprot.org and data was analyzed by MaxQuant (version 1.6.14.0,). The identification FDR cutoffs were set at 0.01 on peptide and protein levels, respectively. The ‘Match between runs’ option was enabled to transfer peptide identifications across RAW files based on accurate retention time and m/z. Quantification was done using a label-free quantification (LFQ) approach based on the MaxLFQ algorithm [24]. A minimum of 2 quantified peptides per protein was required for protein quantification.

The statistical analysis was performed with the R-package “limma” [25]. The LFQ values were normalized via quantile normalization. Adapted from the Perseus recommendations, protein groups with non-zero intensity values in 70% of the samples of at least one of the conditions were used and applied for the imputation of randomly drawn values from a downshifted (1.8 standard deviation) and narrowed (0.3 standard deviation) intensity distribution of the individual sample [26]. The *p*-values were adjusted with the Benjamini–Hochberg method for multiple testing [27].

The LFQ values were used for further downstream processing and differential expression analysis. Proteins were considered as differentially expressed with a log_2_FC ≥ 0.6 (upregulated) or ≤  − 0.6 (downregulated) and an adjusted *p*-value < 0.05. Based on these data the pathway and functional annotation analysis was performed using Ingenuity pathway analysis (IPA, Qiagen) and the Database for Annotation, Visualization and Integrated Discovery (DAVID, v6.8, https://david.ncifcrf.gov/home.jsp), respectively. Data is available from the authors upon request and is publicly available via https://www.ebi.ac.uk/pride/archive/projects/PXD044780/ [28].

### Copy number profiling

gDNA was extracted as described and next-generation sequencing was performed at the DKFZ core facility using two lanes of a NovaSeq SP with 150 PE. Sequenced reads were aligned to the human reference genome using bwa [29] and alignments were sorted and indexed using samtools [30]. Copy-number profiles were generated using Delly’s cnv subcommand [31] with fragment-based GC correction at all short-read mappable positions in the GRCh37 genome. Because of the low coverage, the read depth window size was set to 50 kbp and the baseline ploidy to two. The read-depth signal was segmented using the DNAcopy Bioconductor package [32] using the “sdundo” method with the undo.SD parameter set to three.

### Genetic dependency data analysis and prediction of chr8p synthetic lethality

Genetic dependencies on p-arm of chromosome 8 (chr8p) copy number (CN) were defined as genes with a more severe fitness defect in cell lines that have low chr8p CN as compared with cell lines with high/normal chr8p CN. Those genetic dependencies were hypothesized to represent genes more essential in genes with low chr8p CN and therefore suggest synthetic lethal treatment opportunities for cancers with a loss-of-chr8p. To quantify the fitness defects, genome-wide CRISPR-Cas9 knockout screening data from the Cancer Dependency Map (DepMap) was utilized. Version 20Q2 data comprised fitness effects represented as CERES scores for about 18,119 protein-encoding genes across 769 distinct cell lines (Achilles_gene_effect.csv). Next, CCLE copy number data (CCLE_gene_cn, DepMap 19Q1) were surveyed for the copy number status of chromosome 8p and combined with the respective gene effect data. For a total number of 527 cell lines, chr8p CN was determined for the entire length of the chromosomal arm and adjusted for CN value and deletion size. A copy number value of − 2.5 was determined, below which the chromosomal arm was scored as deleted resulting in chr8p loss/deletion (DEL; *n* = 89) and chr8p high (nonDEL; *n* = 438) classified cell lines. To focus on those of the 18,119 genes that are most likely to be actionable, we downloaded the CCLE expression dataset, which covers 764 of the 769 cell lines screened in the 20Q2 DepMap release (CCLE_expression_v2.csv, https://depmap.org/portal/download/all/?releasename=DepMap+Public+20Q2&filename=CCLE_expression_v2.csv), and only considered genes for the subsequent analysis if their mRNA expression levels had a mean log2 (TPM + 1) larger 1 in the chr8p DEL or the chr8p nonDEL set of cell lines. For each gene, differential CERES (dCERES) scores were computed by subtracting the mean CERES score for that gene in chr8p nonDEL from the mean CERES score for that gene in chr8p DEL. A negative dCERES score therefore indicated that a gene was more essential in chr8p DEL cell lines. The dCERES score presented the differential fitness effect size. To estimate the confidence of the differential effect, *p*-values were computed for each gene using a Wilcoxon Rank Sum test between the DEL and nonDEL cell line CERES scores, using the wilcox.test R function. To reduce false positive hits, Independent Hypothesis Weighting (IHW) was used to adjust *p*-values, using mean CERES scores of chr8p nonDEL cell lines as covariates (also see Additional file 1: Fig. S1a–d). The ihw function from the IHW R package (version 1.26.0) was used to compute the adjusted *p*-values, using an FDR control level of 0.1 [33]. We selected a lenient set of significant effects at an adjusted *p*-value of 0.25 and then prioritized 48 genes with moderately high differential effects (dCERES <  − 0.075) describing a higher essentiality in chr8p DEL cell lines. While we discovered strict effects such as SLC25A28 at an adjusted *p*-value of 0.1, we expanded the hit list for functional characterization of also moderate effects.

To systematically test if the predicted 48 gene dependencies of chr8p were linked to chr8p AND were potential treatment opportunities, we systematically predicted gene–gene buffering using our BaCoN pipeline. In brief, this was done under the assumption that if two genes buffer each other, gene 1 becomes more essential if the expression of gene 2 is downregulated. Therefore, we leveraged the gene fitness effect DepMap data (20Q2) and compared it to the Cancer Cell Line Encyclopedia (CCLE) mRNA expression data of all matched 20Q2 DepMap cell lines. To avoid spurious gene–gene relations, genes were only considered in a potential buffering pair if the potentially buffered gene had sufficient variation in their gene fitness effect (*n* = 3168) and the potentially buffering genes had sufficiently high gene expression (*n* = 3853). Potentially buffering genes had a gene expression standard deviation across all cell lines larger than 1, as well as an expression level > 3 in at least 100 cell lines. Potentially buffered genes had a minimal expression of mean log2 (TPM + 1) > 2 and either an absolute mean CERES > 0.3 or a CERES score standard deviation > 0.2 across all cell lines. For each gene, a Pearson’s correlation coefficient (PCC) of the expression and CERES scores was computed, using the cor function in R. The resulting matrix contains correlation coefficients between each the gene expression of the 3853 potentially buffering genes of the expression set and the CERES scores of each of the 3168 potentially buffering genes of the CERES set. In total, correlations for 12,206,304 gene pairs were computed. To increase the specificity of gene–gene relations, BaCoN weights the PCC of each pair based on the number of higher PCCs of the contributing genes with other genes, divided by the number total number of possible connections of this gene pair. This unsupervised correction of correlation coefficients accounts for biases in cancer cell line CRISPR screening data such as tissue of origin and associated sources of false positive predictions caused by co-expression patterns of buffering genes (Additional file 1: Fig. S1e). The resulting gene pairs were sorted by their weighted PCCs and filtered for pairs including one of the 48 previously defined chr8p genetic dependencies. As pan-DepMap essential genes are not of interest, gene pairs involving a gene with a mean CERES <  − 0.75 across all cell lines were removed.

### Immunohistochemistry

IHC was performed as previously described [17]. Briefly, deparaffinization was achieved by incubating slides in xylene, followed by rehydration using a descending alcohol series and a washing step using water. To enable antigen retrieval, slides were boiled in a pressure cooker for 10 min using a sodium citrate buffer (10 mM Trisodium citrate dihydrate, 0.5%(v/v) TWEEN® 20, pH 6.0), followed by 5 min cool down by washing slides under running water. To block endogenous HRP, the slides were subsequently incubated in 3% hydrogen peroxide for 10 min followed by 1 min washing under running water and twice with PBS for 2 min respectively. The tissue sections were blocked with 5% BSA in PBS with 0.05% Triton X-100 for 1 h at RT and incubated with the primary antibody diluted in blocking buffer overnight at 4 °C (chicken polyclonal anti-GFP, abcam, ab13970, 1:500). Then, slides were washed three times with PBS/Triton X-100 (0.05%) for 5 min and incubated with ImmPRESS® HRP Horse Anti-Rabbit IgG Polymer Detection Kit, Peroxidase (Vectorlabs) for 30 min at RT, followed by three washing steps with PBS/Triton X-100 (0.05%). To allow GFP signal detection, slides were stained with ImmPACT DAB Peroxidase (HRP) Substrate (Vectorlabs) according to the manufacturer’s instructions, and the staining reaction was observed until a sufficiently high staining intensity was achieved. For higher contrasting, a counterstaining was performed by incubating slides in hematoxylin for 1–2 min. After subsequent washing under tap water to sufficiently remove the counterstaining solution, the slides were dehydrated using an ascending alcohol series ending with Xylol and mounted using Surgipath Micromount Mounting Medium (Leica). Histological slides were scanned using the Hamamatsu NanoZoomer Digital Pathology (NDP) system and analyzed using QuPath (Quantitative Pathology & Bioimage Analysis).

### Tissue microarray (TMA) analysis

Samples of primary human tissue were analyzed using TMAs, comprising liver cancer-specific and non-cancerous tissue punches. The liver-specific TMA comprised a total number of 677 tissue punches from human biopsies distributed on 7 slides with 40 histological normal and 178 cirrhotic liver samples as well as 459 HCCs. The histological grading was performed by an experienced pathologist. The individual histochemical MFRN1 staining was quantified using a score ranging from 0 to 4 (MFRN1-Score), with 0 corresponding to no staining and 4 corresponding to very intense staining. The individual MFRN1-Score was assessed quantitively (number of stained cells) and qualitatively (staining intensity and localization). The TMA of healthy human tissue contained 89 tissue punches of 31 different tissue samples and was analyzed similarly. Institutional Ethical Review Board approval was obtained at local Ethical Committees of the Medical Universities of Heidelberg (S205-06), in compliance with the Helsinki Declaration. Written informed consent was obtained from all individuals.

### Xenograft subcutaneous injection of immunodeficient mice

Subcutaneous injection of human and murine tumor cell lines was performed in NSG or Foxn1 nu/nu mice (Janvier) depending on the cell line of origin. A number of 1 × 10^6^ experimentally manipulated cells resuspended in 100 µl PBS harboring DOX inducible shRNA constructs were injected subcutaneously under the right and left flank of isoflurane sedated mice. The tumor volume was assessed by measuring xenograft length (L) and width (W) with skinfold caliper and approximating the three-dimensional volume as (V) = L*(W^2^)/2. After reaching a tumor volume of 100 mm^3^, the mice were given DOX-containing food (Envigo Teklad), and tumor size was periodically measured. Once xenografted tumors reached a diameter of 1.5 cm or ulcerations were observed, animals were euthanized by cervical dislocation. The tumors were resected and photographed using a stereomicroscope (Leica, MZ10F, MC170), tissue samples were flash frozen, and the remaining sample was fixed in 4% paraformaldehyde to be processed for IHC. All animal experiments were conducted in compliance with the regional regulations and with the approval of the regional board in Karlsruhe, Germany (G-81/20).

### Statistics

If not indicated otherwise, statistical analyses were performed using GraphPad Prism 8. If not indicated differently, graphs depict the mean value and standard deviation (SD) of three independent replicates. The significance levels are depicted as *p* < 0.0001 = **** (extremely significant); *p* < 0.001 = *** (extremely significant); *p* < 0.01 = ** (very significant); *p* < 0.05 = * (significant); *p* ≥ 0.05 = ns (non-significant).

## Results

### Predicting the MFRN1-2 paralog synthetic lethality as a vulnerability of chr8p deleted cancers

**Fig. 1:**
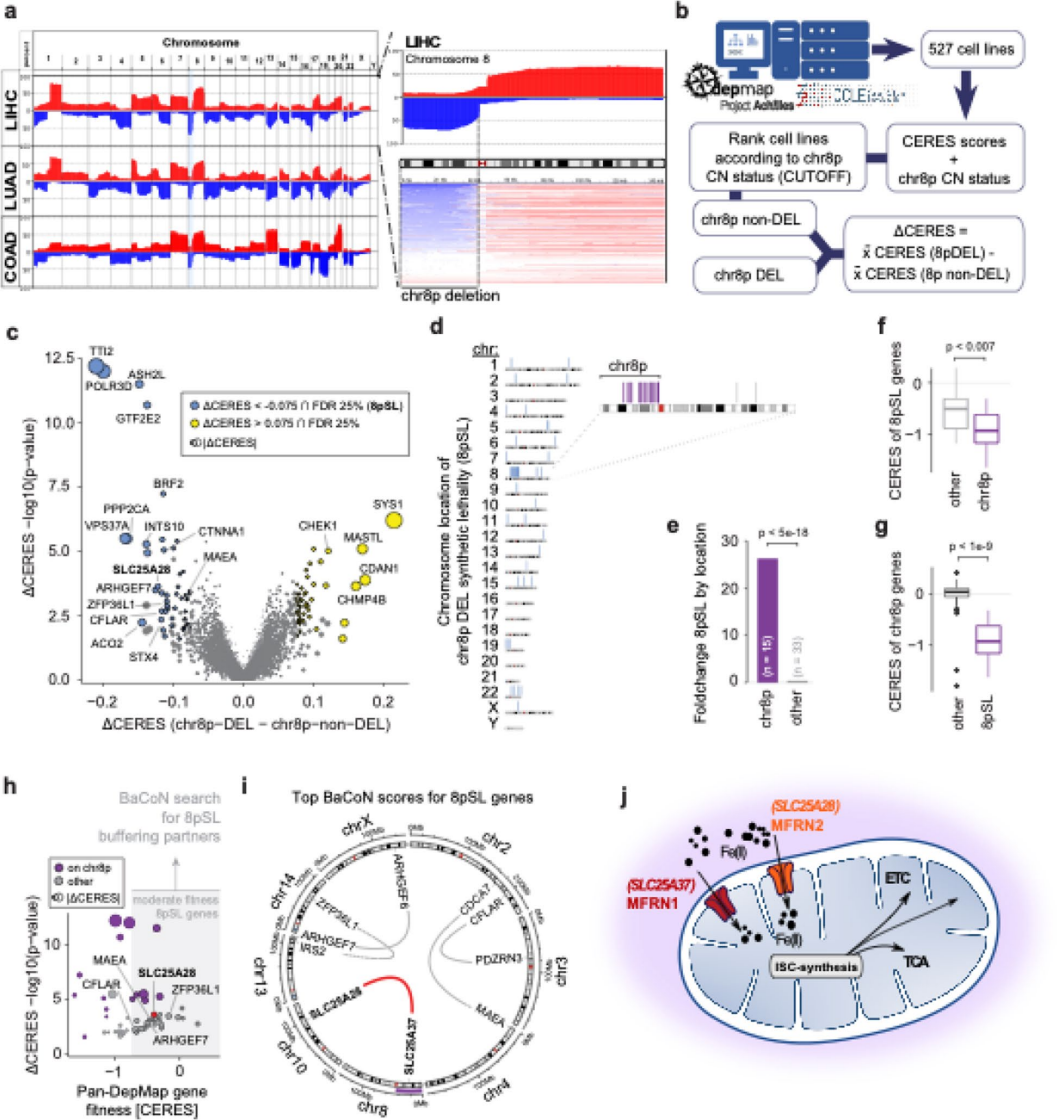
Identification of MFRN1 and 2 paralog synthetic lethality as a specific vulnerability of chromosome 8p deleted cancer cells. **a** Genome view of recurring patterns of SCNAs between tumor entities from publicly available TCGA datasets (LIHC: *n* = 372; LUAD: *n* = 566, COAD = 276). The data is depicted as the number of samples in % with log2(cn/2) > 0.1 for amplifications (red) or the number of samples in % with log2 (cn/2) < 0.1 for deletions (blue) (LIHC: Hepatocellular carcinoma; LUAD: Lung adenocarcinoma; COAD: Colon adenocarcinoma). **b** Simplified overview of the DepMap data analyses workflow to identify genetic vulnerabilities of chr8p deleted cancer cells. **c** Genetic vulnerabilities in chr8p deleted (DEL) versus ch8p non-deleted (non-DEL) cell lines. The vulnerabilities represent the differential cell fitness effects (CERES scores) and are tested for significance using Wilcoxon’s rank-sum test with ISW multiple hypothesis correction (see methods). Significant vulnerabilities (chr8p synthetic lethalities; 8pSL) are defined at an FDR of 25% and a dCERES score <  − 0.075. **d** Chromosomal location for all 48 candidate 8pSL. Chr8 is expanded and 8pSL mapping onto chr8p are highlighted in purple. **e** Enrichment (foldchange) of 8pSL located on chr8p (*n* = 15) compared to 8pSL located in other regions (*n* = 33). Significance was tested using a hypergeometric test. **f** Fitness effects of 8pSL genes located on chr8p (purple) compared to 8pSL genes located elsewhere (gray). Fitness effects represent the per-gene mean CERES score across non-chr8p-DEL cell lines. Significance was tested using Wilcoxon’s rank-sum test. **g** Fitness effects of genes located on chr8p that were identified as 8pSL (purple) compared to non-8pSL genes (grey). Fitness effects represent the per-gene mean CERES score across non-chr8p-DEL cell lines. Significance was tested using Wilcoxon’s rank-sum test. **h** Estimation of dependencies for moderate fitness effect 8pSL genes. 28 (of 48) 8pSL genes had a mean CERES score in chr8p-non-DEL cell lines larger − 0.75. For those genes, the dependency of their fitness effects on the expression of any gene in the genome was tested (see methods for details). Chr8p 8pSL genes are purple. **i** Top 5 dependencies for moderate fitness 8pSL genes (see Table S1 for full list). Circos plot is used to reveal associations between chromosome locations. **j** Schematic overview of the role of MFRN1 and MFRN2. MFRNs are involved in the import of iron into mitochondria where it is used for the synthesis of iron-sulfur clusters (ISC). ISCs act as essential prosthetic groups in several proteins of the ETC and the TCA, as well as other metabolic proteins. ETC, electron transport chain; TCA, tricarboxylic acid cycle

Somatic copy number alteration (SCNA) patterns can be characteristic of specific cancers and pose a putative treatment opportunity. To systematically investigate SCNAs, we mined and queried publicly available sequencing data (TCGA dataportal) from various human solid cancer types (Fig. 1a). Our analysis showed that SCNAs accumulate in specific regions of the genome comprising recurrent patterns across different cancer entities. A prominent instance was the loss of chromosome 8p copy number (Fig. 1a), previously identified as an early event in LIHC [34], pointing to cancer dependencies on genetic elements in this genomic region [4]. Consequently, we focused our attention on chromosome 8p deletions.

To identify potential synthetic lethal targets for 8p deleted cancer cells, we developed an in silico analysis strategy by integrating publicly available high-throughput whole genome CRISPR/Cas9 screening data from the Cancer Dependency Map (DepMap) and matched copy number data (CCLE portal) (Fig. 1b). To increase statistical power, we leveraged the data from cell lines regardless of the tissue of origin. We stratified cell lines into chr8p-deleted and chr8p-non-deleted groups and tested for every gene in the genome for differential cell fitness effects between those groups assigning an effect size and significance measure to each gene (Additional file 1: Fig. S1a–d; see the “ Methods” section for details). We identified 48 genes with a significantly stronger cell fitness defect in chr8p deleted cells, hereafter referred to as chr8p synthetic lethalities (8pSL) (Fig. 1c). Notably, about one third of those genes mapped to chr8p itself (hypergeometric *p*-value < 5e − 18; Fig. 1d, e). Those 8pSL genes on chr8p showed strong cell fitness effects (median CERES =  − 0.92) in chr8p non-deleted cell lines already, which is comparable to the core essential gene set [35, 36] and more severe than non-8pSL genes (Fig. 1f). Moreover, those 8pSL genes on chr8p were more essential than non-8pSL genes on chr8p (Fig. 1g), together suggesting a dosage effect of chr8p essential genes upon partial loss-of-chr8p.

To avoid identifying genes with such an essential gene dosage effect and predict chr8p-linked 8pSL, we applied our *Ba*lanced *Co*rrelation *N*etwork (BaCoN) method on genes with moderate fitness effects (chr8p non-DEL CERES >  − 0.75) (Fig. 1h). BaCoN estimates fitness effect dependency in the DepMap on expression of any other gene in the genome while deprioritizing biases such as cell line tissue of origin in an unsupervised fashion (see methods for details). This strategy enabled us to identify a single 8pSL gene, *SLC25A28* (also known as MFRN2), which is located on chromosome 10, as the SL partner for its chr8p-localized paralog gene *SLC25A37* (also known as MFRN1) (Fig. 1i, Table S1). MFRN1, as well as their paralog MFRN2, are transmembrane transporter proteins, which are involved in shuttling iron into the mitochondria, where they serve pivotal roles in iron-sulfur cluster (ISC) biogenesis and the electron transport chain [14] (Fig. 1j). Together, our in silico analysis revealed MFRN2 as a potential SL candidate in chr8p deleted cancer cells and pointed to paralog synthetic lethality between MFRN1 and MFRN2 as a potential underlying mechanism.

### Genetic epistasis experiments validate MFRN1/2 paralog synthetic lethality as a vulnerability of 8p deleted cancer cells

**Fig. 2:**
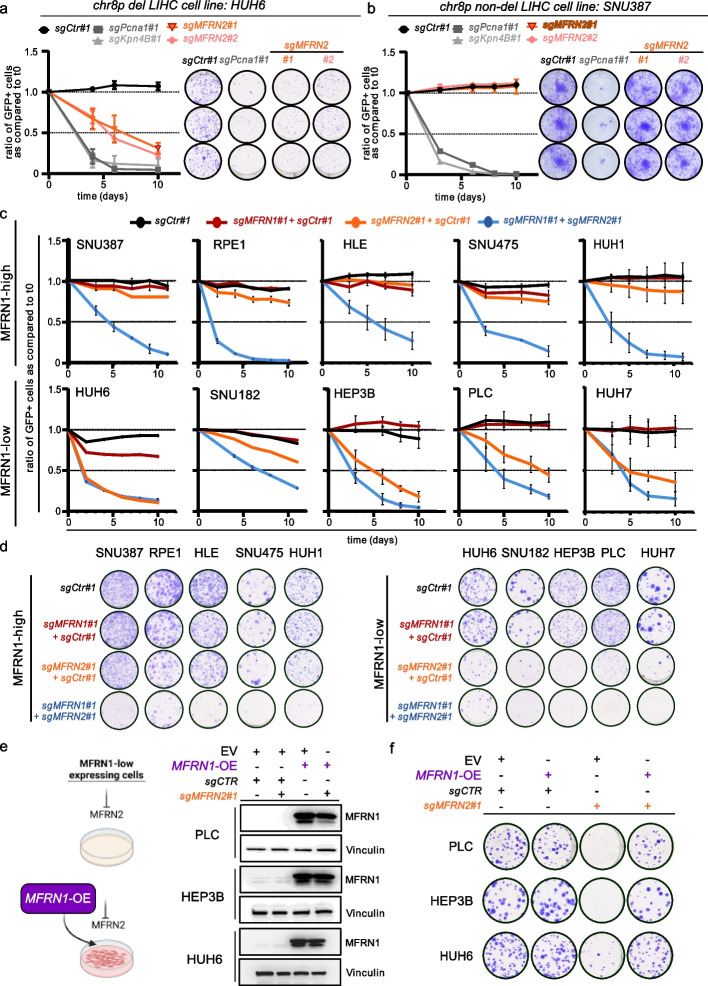
Genetic epistasis experiments validate MFRN1-2 paralog synthetic lethality as a vulnerability of 8p deleted cancer cells. **a**, **b** Competition and Colony formation assays performed in chr8p deleted HUH6 (**a**) and chr8p non-deleted SNU387 liver cancer cell lines (**b**) using two independent sgRNAs to target *MFRN2*. sgRNAs against essential genes *KPNB1* and *PCNA* (gray) were used as positive controls, while a non-targeting sgCTR (black) was used as a neutral control. Competition assay results are shown as the mean ± SD of the ratio of GFP + cells compared to t0 for each assay using FACS analyses over 12 days with measurement at indicated time points. *n* = 3 biological replicates. Colony formation assays are depicted with representative technical triplicates of cells plated into 12-well plates and grown for 12 days *n* = 3 biological replicates. **c** Competition assays of MFRN1 high- and low-expressing cell lines. SpCas9 competent cell lines expressing either a non-targeting sgRNA (sgCTR) or a sgRNA targeting MFRN1 (sgMFRN1) were mixed with cells transduced with a lentiviral construct expressing either a non-targeting sgRNA (sgCTR) or a sgRNA targeting MFRN2 (sgMFRN2) together with GFP in a 30:70 ratio and followed by FACS over 12 days with measurement at indicated time points. Results are shown as the mean ± SD of the ratio of GFP + cells compared to t0 for each assay. *n* = 3 for biological replicates. **d** Colony formation assay corresponding to **c**. *n* = 3 biological replicates. **e** Schematic depicting the workflow of enforced re-expression of MFRN1 in MFRN1-low expressing cells and immunoblot analysis of whole cell lysates from SpCas9 expressing PLC, HEP3B, and HUH6 cells transfected with the indicated lentiviral expression constructs and probed for MFRN1. Vinculin was used as a loading control. Representative results are shown. *n* = 2 biological replicates. **f** Colony formation assay of PLC, HEP3B, and HUH6 cells analyzed in. Cells were seeded in 6-well plates in triplicates and cultured for 14 days. *n* = 3 biological replicates

To functionally validate the predicted SL of MFRN2 and MFRN1 we performed gene-targeting experiments in human cancer cell lines. We first focused on liver cancer cell lines given the frequency of chr8p deletions in this cancer entity [4] (Fig. 1a). We selected cell lines based on their chr8p deletion status as well as their MFRN1 transcript and protein expression status and grouped them into MFRN1 high or low groups (Additional file 1: Fig. S2). We then infected viral vector constructs encoding for sgRNAs linked to GFP into two hepatocellular carcinoma cell lines, HuH6 (chr8p-deleted) and SNU387 (chr8p-proficient), expressing Cas9 and performed cell competition assays as well as colony formation assays (CFA) (Fig. 2a, b). Whereas positive controls targeting essential genes (*PCNA* and *KPNB1*) strongly impaired fitness in both cell lines, we found that CRISPR/Cas9-mediated perturbation of *MFRN2* reduced proliferation only in HuH6 cells (8p-deleted) and had no effect in SNU387 cells (8p-proficient) (Fig. 2a, b). Notably, of nine additional chr8pSL genes suggested from our initial pre-filtering analysis (Fig. 1c) only two, *Ash2l* and *Aco2*, reproduced the specific discriminative lethality pattern observed for *MFRN2* when CRISPR/Cas9 constructs were used for gene perturbation in chr8p-deleted versus chr8p-non-deleted cell lines (Additional file 1: Fig. S3). Thus, utilizing various filtering steps and integration of multiple datasets to identify SL interactions from DepMap seemingly aids in pinpointing SL targets for specific chromosomal deletions.

To directly dissect the paralog synthetic lethal relationship of *MFRN1* and *MFRN2*, we next designed potent sgRNAs targeting each gene (see Additional file 1: Fig. S3a for guide validation) and used them for genetic epistasis experiments in both, MFRN1-high and MFRN1-low liver cancer cell lines by cell competition assays (Fig. 2c). Whereas in MFRN1-high cells only simultaneous deletion of *MFRN1* and *MFRN2* led to a competitive growth disadvantage, this phenotype was already achieved by disrupting *MFRN2* alone in MFRN1-low cells (Fig. 2c). CFAs confirmed these observations (Fig. 2d). To further demonstrate this functional relationship, we used human liver cancer cell lines with low MFRN1 expression and ectopically re-expressed MFRN1 (Fig. 2e). Strikingly, MFRN1 re-expression completely abolished the detrimental effect of MFRN2 deletion in these cell lines (Fig. 2f). Using an orthogonal RNAi-mediated approach to perturb MFRN2 expression corroborated our findings (Additional file 1: Fig. S4). To test if the relationship between MFRN1 and MFRN2 can be observed beyond liver cancer cells, we analyzed MFRN1 expression in colon and lung cancer cell lines (Additional file 1: Fig. S5). We selected relevant human cancer cell lines with high or low MFRN1 expression and performed shRNA-mediated *MFRN2* perturbation experiments to investigate its impact on cell growth in dependence on MFRN1 protein expression. Similar to liver cancer cell lines, *MFRN2* knockdown impaired cell growth specifically in colon and lung cancer cell lines with low MFRN1 expression (Additional file 1: Fig. S4).

Taken together, these results demonstrate that MFRN2 dependency is dictated by MFRN1 proficiency and functionally proof paralog synthetic lethality as the underlying mechanism in chr8p deleted cancer cells.

### Targeting MFRN2 provides a vulnerability that correlates with MFRN1 expression

**Fig. 3:**
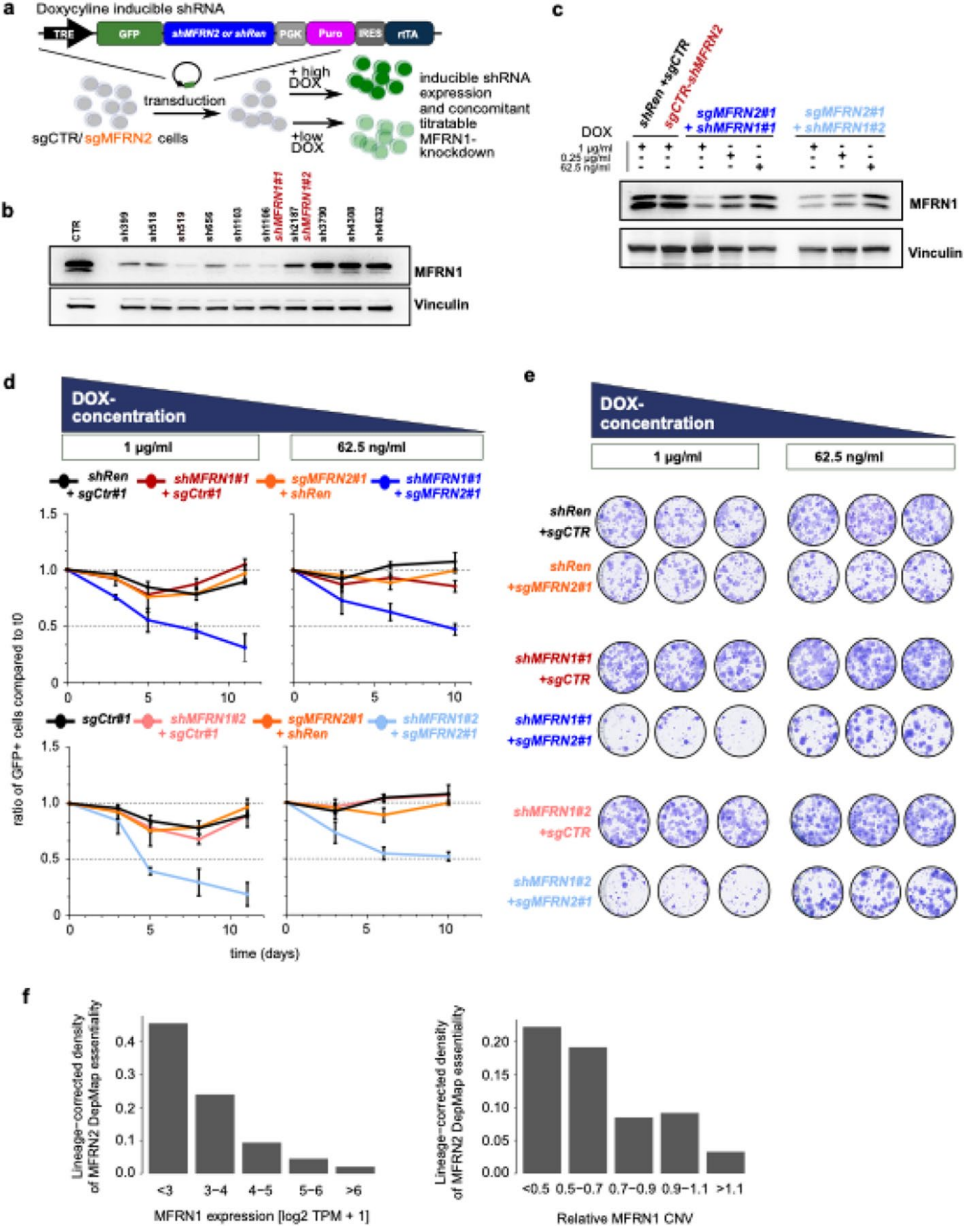
Targeting MFRN2 provides a vulnerability that correlates with MFRN1 expression. **a** Schematic of the Doxycycline (DOX)-inducible lentiviral shRNA expression constructs used. Tet-regulated vectors expressing GFP-coupled miRE shRNAs and rtTA as a Tet-On all-in-one construct. Lentiviral transduction and supplementation with DOX leads to expression of GFP as well as the expression of the individual shRNA-cassette. **b** Knockdown efficiency of shRNAs targeting MFRN1. Immunoblot analysis of MFRN1 using whole cell protein lysates from HLE cells transfected with retroviral constitutive expression constructs harboring the indicated shRNAs probed for MFRN1. A shRNA targeting Renilla luciferase was used as control (CTR). Marked in red are the shRNAs selected for experiments. Vinculin served as loading control. Representative results are shown. *n* = 1 biological replicates. **c** Knockdown titration experiments. Immunoblot analysis of MFRN1 using whole cell protein lysates from HLE cells transduced with the indicated plasmid combinations and cultured in medium supplemented with the 1 μg/ml, 0.25 μg/ml, or 62.5 ng/ml doxycycline (DOX) to titrate shRNA expression. Representative results are shown. *n* = 2 biological replicates. **d** Competition Assays of HLE cells transduced with the indicated plasmids and cultured in medium supplemented with the 1 μg/ml or 62.5 ng/ml DOX. The individual cell lines were mixed in a ratio of 30:70 with cells harboring an MFRN2 KO and no GFP expression. Results are shown as the mean ± SD the ratio of GFP + cells compared to t0 for each assay performed with 3 technical replicates. Representative of 1 biological replicate is shown. *n* = 2 for biological replicates. **e** Colony formation assay corresponding to **d**. *n* = 2 biological replicates. **f** Dependency of MFRN2 essentiality across 764 cell lines in the Cancer Dependency Map (DepMap, 20Q2) on MFRN1 expression and copy number variation (CNV) levels. The cell line lineage effect was regressed out using multiple linear regression and MFRN2 essentiality was defined as the lowest 10% of lineage-corrected CERES scores

*MFRN1* is predominantly heterozygous deleted (64%) and only 8% of the samples reveal homozygous deletions (Fig. 1a and Additional file 1: Fig. S2c), suggesting incomplete MFRN1 loss. Hence, we directly modeled reduced MFRN1 expression by leveraging doxycycline-inducible shRNAs against *MFRN1* alongside Cas9-mediated perturbation of *MFRN2* (Fig. 3a, b) to investigate if reduced MFRN1 expression was indeed sufficient to establish a synthetic lethal relationship to its paralog MFRN2. This approach allowed us to titrate MFRN1 protein expression in a doxycycline dose-dependent manner in cells that otherwise exhibited high MFRN1 protein expression (Fig. 3c). We observed a competitive disadvantage for MFRN2 deleted cells upon *MFRN1* knockdown, which was dose-dependent on MFRN1 protein expression levels (Fig. 3d, e). We were able to validate this dose-dependent relationship in data derived from loss-of-function screens in approximately 500 cancer cell lines from different lineages (DepMap portal). After correcting for the lineage effect, we found that lower *MFRN1* transcript expression and copy number coincided with higher *MFRN2* dependency (Fig. 3f). Thus, even dosage reduction of MFRN1 will lead to reduced cellular fitness upon MFRN2 inhibition.

### Targeting MFRN2 in a MFRN1-deficient background affects iron - dependent mitochondrial processes

**Fig. 4:**
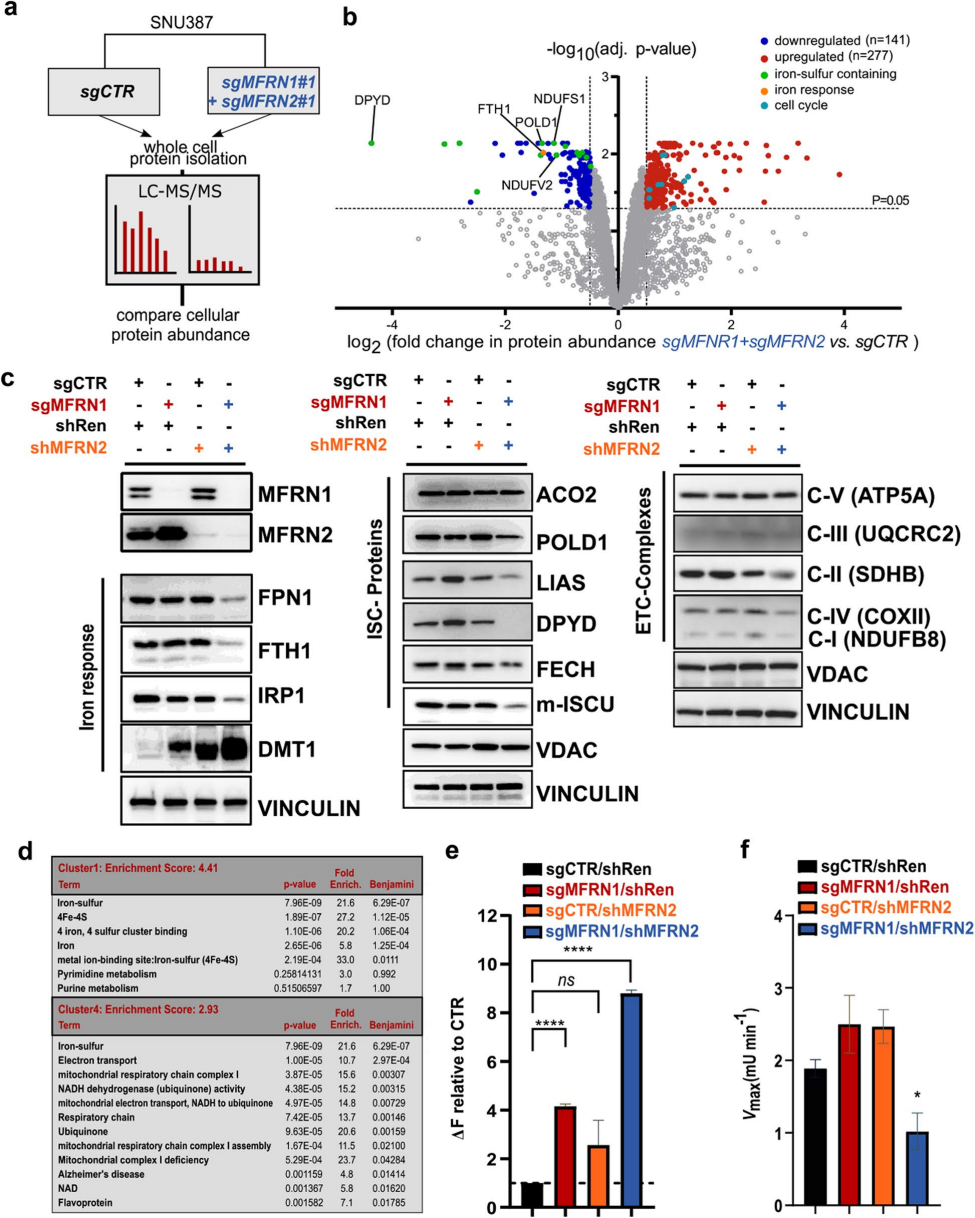
Targeting MFRN2 in a MFRN1-deficient background affects iron-dependent mitochondrial processes as identified by unbiased proteomics. **a** Simplified overview of the workflow used for sample preparation for proteome analysis performed from whole cell protein lysates of SNU387cells with no control sgRNAs (sgCTR) or combined CRISPR/Cas9-mediated MFRN1/MFRN2 KO. **b** Volcano plot showing the relative fold change (log2) in protein abundance versus -log (adj. *p*-values) from SNU387 cultured for 6 days, harboring a simultaneous KO of MFRN1 and MFRN2 or no KO (CTR). Proteins were considered as differentially expressed with a log2FC ≥ 0.6 (upregulated; red data points) or ≤  − 0.6 (downregulated, blue data points) and an adjusted *p*-value < 0.05. The dotted line represents the adjusted *P* = 0.05. Colors denote indicated protein family or pathway affiliation. *n* = 3 biological replicates. **c** Immunoblot analysis of essential proteins involved in the iron response pathway, ISC-synthesis, and ETC-complex. Cytosolic and mitochondrial fractionated cell lysates from SNU387 and PLC cells were probed for the indicated proteins after 6 days of culture in a DOX-containing medium. Vinculin served as a cytosolic, VDAC as a mitochondrial loading control. *n* = 2 biological replicates. ETC, electron transport chain; ISC, iron-sulfur cluster. **d** DAVID functional annotation clustering of downregulated proteins showing terms containing at least 3 detected proteins with Benjamini–Hochberg adjusted *p* > 0.05. Fold enrichment relative to the *H. sapiens* proteome is displayed. **e** Measuring the labile iron pool (LIP) with Calcein assays. SNU387 cells were targeted for MFRN1 KO, MFRN2 KD, or simultaneous MFRN1-KO/MFRN2-KD with the indicated sgRNA/shRNA combinations. shRenilla (shRen) was used as a control shRNA. Cells were cultured for 6 days in a DOX-containing medium before staining with Calcein-AM and FACS readout. One day prior to measurement, cells were split and either treated with the iron chelators Ciclopirox (CPX) and Deferoxaomine (DFO) or left untreated. To determine the difference in LIP, media of CPX/DFO treated samples was subtracted from the non-treated samples (ΔF). Results are shown as mean + SD. *n* = 3 biological replicates. Statistical significance was determined by a two-tailed, two-sample *t*-test (****p < 0.0001, ***p < 0.001). **f** Aconitase activity measurement (Vmax) from isolated mitochondrial lysates of SNU387 after 6 d and treatment as described above. mU = milli units. Results are shown as mean + SD. *n* = 2 biological replicates. Statistical significance was determined by a two-tailed, two-sample *t*-test (*p < 0.05, ns = non-significant)

To understand the mechanistic underpinnings of the paralog synthetic lethal relationship between MFRN1 and MFRN2, we next performed unbiased proteomics and compared parental cell lines with their combined MFRN1 and 2 knockout counterparts (Fig. 4a). We observed a dramatic change in the proteome of MFRN1/2 perturbed cells, with downregulation of numerous proteins (*n* = 141), many of which contained iron-sulfur clusters, such as POLD1 and DPYD (Fig. 4b). Immunoblot and pathway analyses confirmed a large fraction of proteins associated with cellular iron response, iron-sulfur cluster containing proteins, and factors involved in mitochondrial oxidative phosphorylation as being downregulated upon combined MFRN1 and 2 loss (Fig. 4c, d) underlining the function of both paralogs as transmembrane transporter proteins involved in shuttling iron into mitochondria. Along this line, we observed an increase in cytosolic iron levels as a consequence of combined MFRN1 and 2 perturbation (Fig. 4e) arguing for impaired iron import into mitochondria and a loss in activity of the ISC-dependent mitochondrial protein Aconitase 2 (Fig. 4f). Thus, these results demonstrate a substantial impact of simultaneous MFRN1 and MFRN2 loss on iron-dependent mitochondrial processes and overall cellular iron homeostasis.

### Targeting MFRN2 in a MFRN1-deficient background disrupts mitochondrial homeostasis and causes cell death

**Fig. 5:**
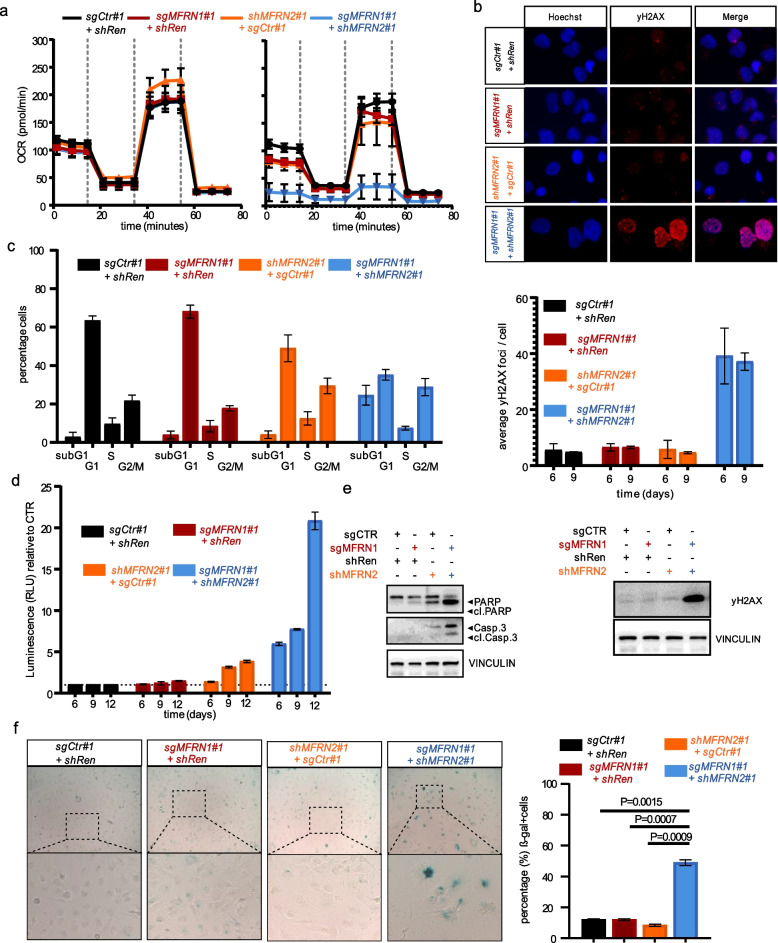
Targeting MFRN2 in a MFRN1 deficient background disrupts mitochondrial homeostasis and causes cell death. **a** Sea horse Mito Stress assay of SNU387 cells targeted with the indicated sgRNA and shRNA combinations. Cells were analyzed for their OCR in a 96-well format before and after the addition of the complex-specific inhibitors. *n* = 2 biological replicates Left: initial time point, right: 6 days post-DOX treatment. **b** Immunofluorescence analysis of yH2AX foci by fluorescence microscopy after treatment of SNU387 cells as described in **a**. Upper panel: Representative images of SNU387 cells with the indicated treatments after 6 days. Primary yH2AX antibody was incubated with Alexa 594-labeled (red) secondary antibody. Nuclear DNA (blue) was counterstained with Hoechst. middle panel: Shown is the mean ± SD of the average yH2AX-foci per cell quantified using ImageJ and counting at least 100 nuclei in 3 biological replicates. Lower panel: Immunoblot analysis of whole cell lysates from SNU387 cells treated as described in **a** and probed for yH2AX. Vinculin was used as a loading control. Representative results are shown. *n* = 2 biological replicates. **c** Cell cycle analysis of SNU387 cells transduced with the indicated plasmid combinations after being cultured in DOX-containing medium for 6 days. FACS analysis was performed at the indicated time points. Results are shown as mean + SD. *n* = 3 biological replicates. **d** Apoptosis assay measuring active Caspase-Glo 3/7 by luminescence measurement at the indicated timepoints in SNU387 cells transduced as described above. Shown is the mean ± SD of the relative luminescence compared to CTR cells (sgCTR + shRen). *n* = 3 biological replicates. **e** Immunoblot analysis of whole cell lysates from SNU387 cells treated as described above and probed for Caspase-3 and PARP-1. Cleaved protein indicates apoptosis. Vinculin was used as a loading control. Representative results are shown. *n* = 2 for biological replicates. **f** Representative images at 40 × magnification showing SA-beta-gal staining of SNU387 cells treated as described above after 6 days of culturing in DOX-containing medium (left panel). Brightfield images were quantified with ImageJ by counting at least 200 cells per condition in 3 independent replicates. Graphs show mean + SD for *n* = 3 biological replicates. Statistical significance determined by a two-tailed, two-sample *t*-test (****p < 0.0001, **p < 0.001, *p < 0.05, ns = non-significant) (right panel)

To further investigate the phenotypic consequences of MFRN1/2 paralog synthetic lethality, we next concentrated on direct measurements of mitochondrial function and overall cellular fitness. In line with the important role of MFRNs in mitochondrial homeostasis MFRN1/2 perturbation had a dramatic impact on respiratory capacity as evidenced by measurement of oxygen consumption rate [37] (Fig. 5a). We further observed that combined MFRN1/2 perturbation in MFRN1-high expressing cells culminated in extensive DNA damage as measured by yH2AX expression (Fig. 5b) and was accompanied by substantial cell cycle defects with an increase in subG1 and G2 phase, the former indicative of cell death, and a decrease in G1-phase cells (Fig. 5c). This finding was corroborated by direct measurements of apoptosis markers, namely Caspase3 activity and PARP cleavage (Fig. 5d, e). Nonetheless, we also observed an increase in senescent cells (Fig. 5f) alongside apoptosis. Similar results were observed in MFRN1 low-expressing cell lines already with MFRN2 perturbation alone (Additional file 1: Fig. S6a–d). Importantly, forced MFRN1 re-expression in MFRN1 low-expressing cell lines completely abolished the observed effects, further demonstrating that phenotypic consequences of MFRN2 inhibition are dictated by MFRN1 protein levels (Additional file 1: Fig. S6e,f). Taken together, these results indicate that depletion of MFRN proteins leads to disruption of mitochondrial homeostasis, and to a multi-facetted detrimental impact on cellular fitness.

### Targeting MFRN2 in a MFRN1-deficient background abolishes tumor growth of liver *cancer* cells in preclinical in vivo experiments

**Fig. 6:**
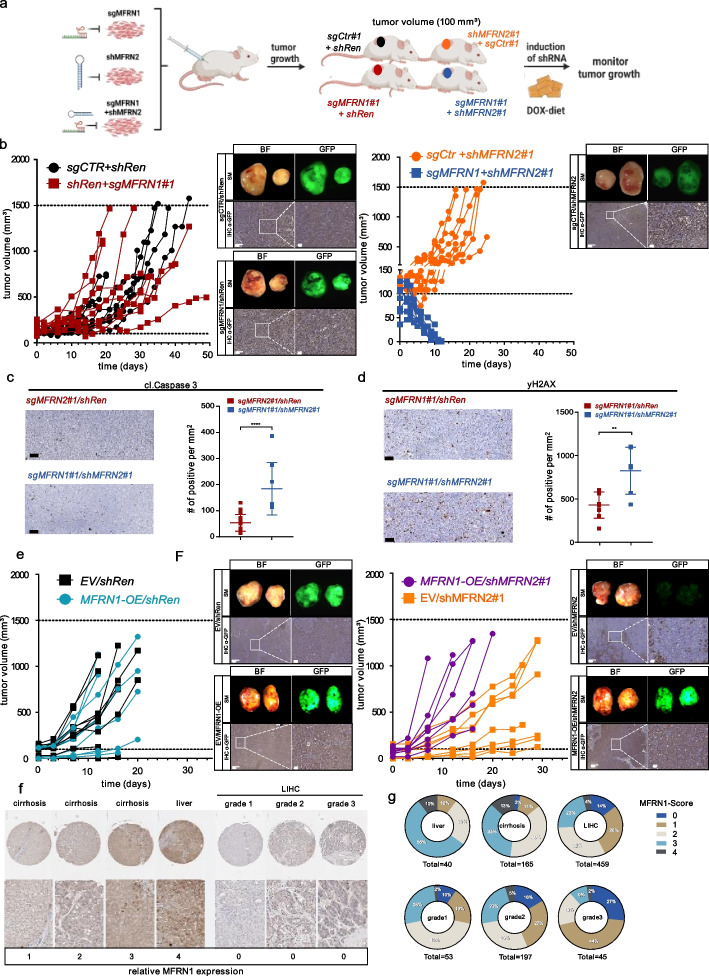
Targeting MFRN2 in a MFRN1-deficient background abolishes tumor growth of liver cancer cells in preclinical in vivo experiments. **a** Schematic illustration of the workflow of human xenograft tumors in immunodeficient NSG mice. MFRN2 or control perturbation was induced once established tumors reached 100 mm^3^ in size. **b** SNU387 cells stably expressing SpCas9 with either sgMFRN1 or sgCTR together with a construct expression of either shMFRN2 or shRenilla (shRen) were sc. injected into NSG nude mice (*n* = 5 female mice per group). Right panel: Stereomicroscopic imaging with macroscopic pictures of exemplary tumors in Brightfield (BF) exposure and GFP-channel. Immunohistochemistry staining of GFP (IHC α-GFP) of the same samples with different magnifications. GFP-positivity indicates shRNA expression. Scalebar: 500 μm and 100 μm. **c** Apoptosis assessment based on Caspase 3 activation. Left panel: Representative images for cleaved Caspase3. Right panel: Quantification of IHC staining for cleaved Caspase3. Time point post-DOX = 7d. Statistical analysis unpaired *t*-test. Scale bar = 100 µm. **d** DNA damage assessment based on yH2Ax expression. Left panel: Representative images for yH2Ax. Right panel: Quantification of IHC staining for yH2Ax. Time point post-DOX = 7 days. Statistical analysis unpaired *t*-test. Scale bar = 100 µm. **e** PLC cells stably expressing SpCas9 with either a construct expressing MFRN1 or a control construct (EV) together with a construct expression of either shMFRN2 or shRenilla (shRen) were sc. injected into NSG nude mice (*n* = 5 female mice per group). Right panel: Stereomicroscopic imaging with macroscopic pictures of exemplary tumors in Brightfield (BF) exposure and GFP-channel. Immunohistochemistry staining of GFP (IHC α-GFP) of the same samples with different magnifications. Scalebar: 500 μm and 100 μm. **f** Representative images of MFRN1 immunohistochemistry in human tissue specimens of normal liver tissue (liver), cirrhotic livers (cirrhosis) or hepatocellular carcinomas (LIHC) of different grading (grade 1–3) in a tissue microarray (TMA). Samples were individually scored (MFRN1-Score) according to their staining intensities as indicated on the left. **g** Donut diagram showing the MFRN1-Score distribution among liver samples. Colors represent the respective MFRN1-Score as indicated in the legend. Numbers within each wedge are given as percentage of the total number of counted samples as indicated below

Encouraged by our cell culture-based experiments we next investigated if the observed paralog lethality can be leveraged to impair tumor growth in vivo. To this end, we performed xenograft experiments with human liver cancer cells genetically modified to harbor Cas9-mediated MFRN1 loss-of-function alongside inducible knockdown of *MFRN2*. Following the establishment of small tumor nodules (100 mm^3^), we induced *MFRN2* knockdown by doxycycline-containing diet and monitored tumor growth over time (Fig. 6a). Strikingly, *MFRN2* targeting completely eradicated subcutaneous MFRN1 KO tumors (Fig. 6b right panel), whereas tumors expanded rapidly in conditions in which we used an inducible shRNA targeting Renilla luciferase as a control perturbation (Fig. 6b, left). Further, *MFRN2* knockdown did not impair tumor growth in MFRN1 proficient parental cancer cells (Fig. 6b). Of note, in a subsequent experiment where we harvested tumors shortly after MFRN2 targeting, we observed that combined MFRN1/2 perturbation was associated with apoptosis, specifically an increased expression of cleaved caspase (Fig. 6c), as well as an increase in DNA-damage markers in tumor tissues (Fig. 6d) thereby corroborating our in vitro results and providing a mechanistic explanation for its marked impact on tumor growth.

Next, using a xenograft model with MFRN1 low expressing human liver cancer cells, we observed that shRNA-mediated *MFRN2* perturbation alone delayed tumor growth, which could be mitigated by forced overexpression of MFRN1 (Fig. 6e). This finding aligns with the observed MFRN1 expression level-dependent reduction in proliferation observed in vitro (Fig. 3d) and lends further support to the direct relationship between MFRN1 expression level and the severity of MFRN2 targeting synthetic lethality. Our findings were further substantiated in cell lines derived from a murine liver cancer model system, in which we observed paralog synthetic lethality for simultaneous Cas9 and/or shRNA-mediated MFRN1/2 perturbation (Additional file 1: Fig. S7a–f) and pronounced tumor growth retardation in xenograft tumors upon inducible *MFRN2* knockdown only in the setting of MFRN1 loss (Additional file 1: Fig. S7g).

Given that our results suggested that MFRN1 expression is the complementary biomarker for MFRN2-directed therapeutic opportunities, we finally aimed to assess the clinical contexts in which specific vulnerability might be exploited. First, we investigated *MFRN1* and 2 transcript expression using publicly available databases [38, 39]. MFRN1 functions as an essential mitochondrial iron importer in developing erythroid cells and, as expected [40], its expression was highest in whole blood (Additional file 1: Fig. S8a). However, MFRN1 expression could also be detected in all other human tissue analyzed. Further, we found that *MFRN2* is expressed in every human tissue type with low levels in whole blood (Additional file 1: Fig. S8a). When comparing mRNA expression of *MFRN1* in tumor versus normal tissue we found that most solid tumor types exhibit lower *MFRN1* expression (Additional file 1: Fig. S8b).

To directly measure MFRN1 protein expression in human tissue samples, we finally established immunohistochemistry (IHC) for MFRN1 detection on a large number of samples from healthy and cancerous tissue (Fig. 6f,g and Additional file 1: Fig. S8c,d). Reassuringly, we generally observed MFRN1 expression in healthy tissue albeit with varying strength, such as high expression in e.g. pancreas and liver and low expression in e.g. prostate and spleen (Additional file 1: Fig. S7c). Focusing on liver cancer, we observed high MFRN1 expression in healthy liver tissue, whereas MFRN1 protein expression was low in cirrhotic livers and more markedly reduced in liver cancer tissue samples (Fig. 6f,g). Of note, we further observed that the fraction of samples with low or no MFRN1 increased with higher tumor grade (Fig. 6f,g). MFRN1 expression was likewise low in a substantial number of solid cancer types of different origins (Additional file 1: Fig. S8d) and its expression was completely absent in 4–33% of the tumor samples analyzed, with lung tumors revealing the highest incidence and liver tumors ranking second with 14% (Additional file 1: Fig. S8d and Fig. 6g).

In summary, our results suggest that MFRN2 targeting could provide an approach to specifically target tumors with low MFRN1 expression and nominate MFRN1 as the complimentary biomarker amenable to IHC-based patient stratification.

## Discussion

Somatic copy number alterations are a hallmark of cancer genomes with significant implications in various tumorigenic processes [3]. Their recurrent patterns across different cancer types make them attractive targets for identifying specific vulnerabilities that can be exploited therapeutically [5]. In this study, we identify and characterize a paralog synthetic lethality relationship between MFRN1 and MFRN2, which can be exploited to specifically target chr8p deleted tumors. Provided that a majority of clinical trials are still conducted on patients lacking biomarkers, which results in missed treatment opportunities [41], it is noteworthy that our study nominates MFRN1 expression status as a complementary biomarker for the potential efficacy of MFRN2-directed therapies, even independent of chr8p-deletions.

Conceptually, our study aligns with previous research emphasizing the value of large-scale functional genomics datasets in identifying vulnerabilities associated with characteristic molecular signatures of tumors [8, 12, 42, 43]. However, our improved synthetic lethality prediction platform predicted paralog gene pairs, which are known to functionally buffer each other, with higher precision. As functional screening technologies, like combinatorial perturbation screening with Cas12a continue to advance [44, 45], and more cancer models provide data [46], the arsenal of therapeutically exploitable targets can hopefully be expanded and solidified. Recent studies using Cas12a-based combinatorial knockout screens and other similar approaches have uncovered the SL relationship between *MFRN1* and *MFRN2* in individual cell lines [47–49], further validating our general, cell line-spanning paralog synthetic lethality prediction in an orthogonal experimental system.

From a biological perspective, our results underscore the redundancy and essentiality of both MFRN paralogs to safeguard mitochondrial iron homeostasis and associated ISC-related processes in mammalian cells. Initially, it was believed that MFRN1 primarily fulfills the increased demand for mitochondrial iron in erythroid cells for heme production, while MFRN2 facilitates iron import in non-erythroid cells [15]. Our results show that MFRN1 and MFRN2 are both expressed in almost every tissue type and that their functional redundancy is crucial to safeguard mitochondrial iron import to guarantee cellular iron homeostasis in cancer cells from various tissues. These results are consistent with findings demonstrating that tissue-specific knockout of MFRN1 and MFRN2 together leads to reduced mitochondrial iron levels and decreased expression of oxidative phosphorylation proteins, whereas their individual depletion does not lead to severe phenotypic effects in hepatocytes under standard housing conditions [40, 50].

From a therapeutic viewpoint, our study highlights the potential of targeting MFRN2 as a specific approach to combat MFRN1-deficient tumors. Notably, while MFRN1 knockout led to embryonic lethality in mice, the deficiency of MFRN2 did not impact animal viability [15, 16, 40], indicating a relative lack of adverse effects from inhibiting MFRN2 on healthy tissue. This finding supports MFRN2 inhibition as a promising therapeutic strategy with potentially few detrimental consequences. Yet, the high degree of homology between MFRN1 and MFRN2 as well as its location within mitochondrial membranes may pose challenges for the pharmacological tractability of MFRN2. Nonetheless, encouraging results with mitochondriotropic small-molecule inhibitors suggest the feasibility to efficiently target mitochondrial ion channels [51]. Alternatively, considering the significant reliance on MFRN2-mediated mitochondrial iron homeostasis in MFRN1-deficient cells, pharmacologically modulating overall iron homeostasis or associated cellular processes itself could provide an opportunity to target these cells. The pronounced chr8-deletion-specific effect of *Aco2* perturbation alongside reduced Aconitase2 activity as a result of MFRN targeting that we observed lends further support to this hypothesis. Additional work probing for the effects of currently available compounds to modulate the intracellular labile iron pool [52, 53] are hence warranted.

The results we obtained in our preclinical models indicated that substantial depletion of both, MFRN1 and MFRN2 can result in tumor eradication. However, since resistance to targeted therapy constitutes a major challenge [54], it remains to be seen if and how cells that are impaired in MFRN activity could bypass this strong dependence on MFRNs for mitochondrial iron import. Notably, alternative mechanisms for iron transport into mitochondria have been proposed already [55, 56] and could in the future provide opportunities to target these adaptive responses [57].

In summary, the paralog synthetic lethal relationship between MFRN1 and MFRN2 revealed by our study presents a pharmacologically accessible Achilles' heel, which can be exploited to selectively target MFRN1-deficient tumors.

In the context of future clinical implementation, it is noteworthy that deletions affecting chromosome 8 typically result in heterozygous changes in MFRN1 copy numbers. This heterozygosity may pose a challenge for MFRN2-targeting therapies, as it suggests that the remaining copy of MFRN1 may render MFRN2 inhibition insufficient to eradicate tumors. Since we demonstrate a direct relation between MFRN1 expression levels and the degree of synthetic lethality provided by MFRN2 perturbation, we anticipate that utilizing MFRN1 immunohistochemistry can provide a straightforward and reliable biomarker to inform about the potential efficacy of MFRN2-targeted therapies.

## Conclusions

Our data reveal MFRN2 as a potential therapeutic target of chromosome 8p deleted cancers and nominate MFNR1 as the complementary biomarker for MFRN2-directed therapies. Further studies are warranted to estimate the druggability of MFRN2.

### Supplementary Information

Additional file 1: Figures S1 – S8.

Additional file 2: Table S1: Contains differential (d)CERES scores for chromosome 8p deleted cell lines and BaCoN SL scores for all significant chromosome 8p dCERES hits.

## Data Availability

All data and code relevant to this work are available from the authors upon request. We deposited mass spectrometry data accompanying our work and made it publicly accessible at https://www.ebi.ac.uk/pride/archive/projects/PXD044780/ [28].
