# Supplementary material for: Mitoferrin2 is a synthetic lethal target for chromosome 8p deleted cancers
Source: Genome Med. 2024 Jun 17;16:83. doi: 10.1186/s13073-024-01357-w (PMC11181659; doi:10.1186/s13073-024-01357-w)
Supplement: Supplementary file 1 — Additional file 1: Figures S1 – S8. [file 13073_2024_1357_MOESM1_ESM.docx]

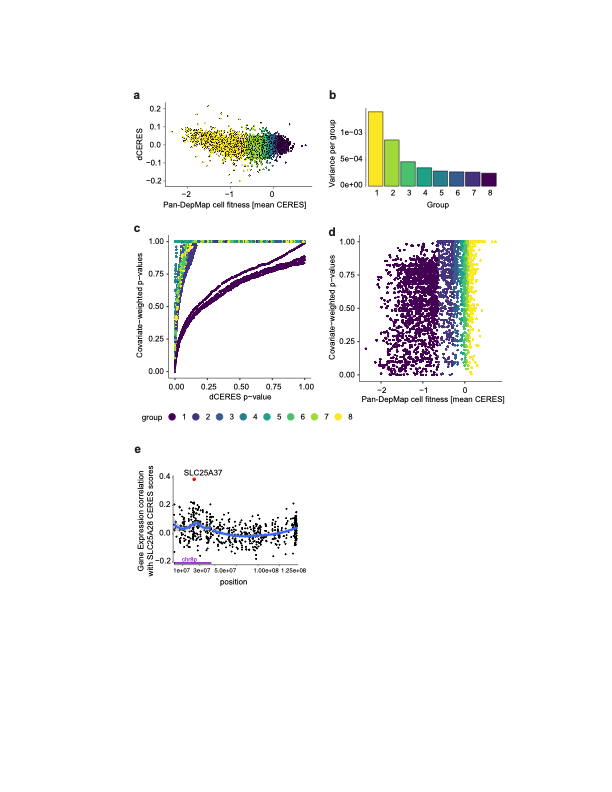


**Fig S1: Prediction of loss-of-chr8p synthetic lethalities**. **a)** Pan-Cancer Dependency Map (DepMap) as a co-variate of chr8p low vs high differential fitness effects (dCERES). Each point represents a gene, each color an IHW-defined group. **b)** dCERES variance in the IHW-defined groups. **c)** Uncorrected (x-axis) versus IHW-adjusted p-values for each gene. **d)** IHW-adjusted p-values versus pan-DepMap cell fitness effects of each gene. **e)** Pearson’s correlation coefficients between SLC25A28 fitness effects in the DepMap and each gene on chr8. The location of the chr8p arm is marked.


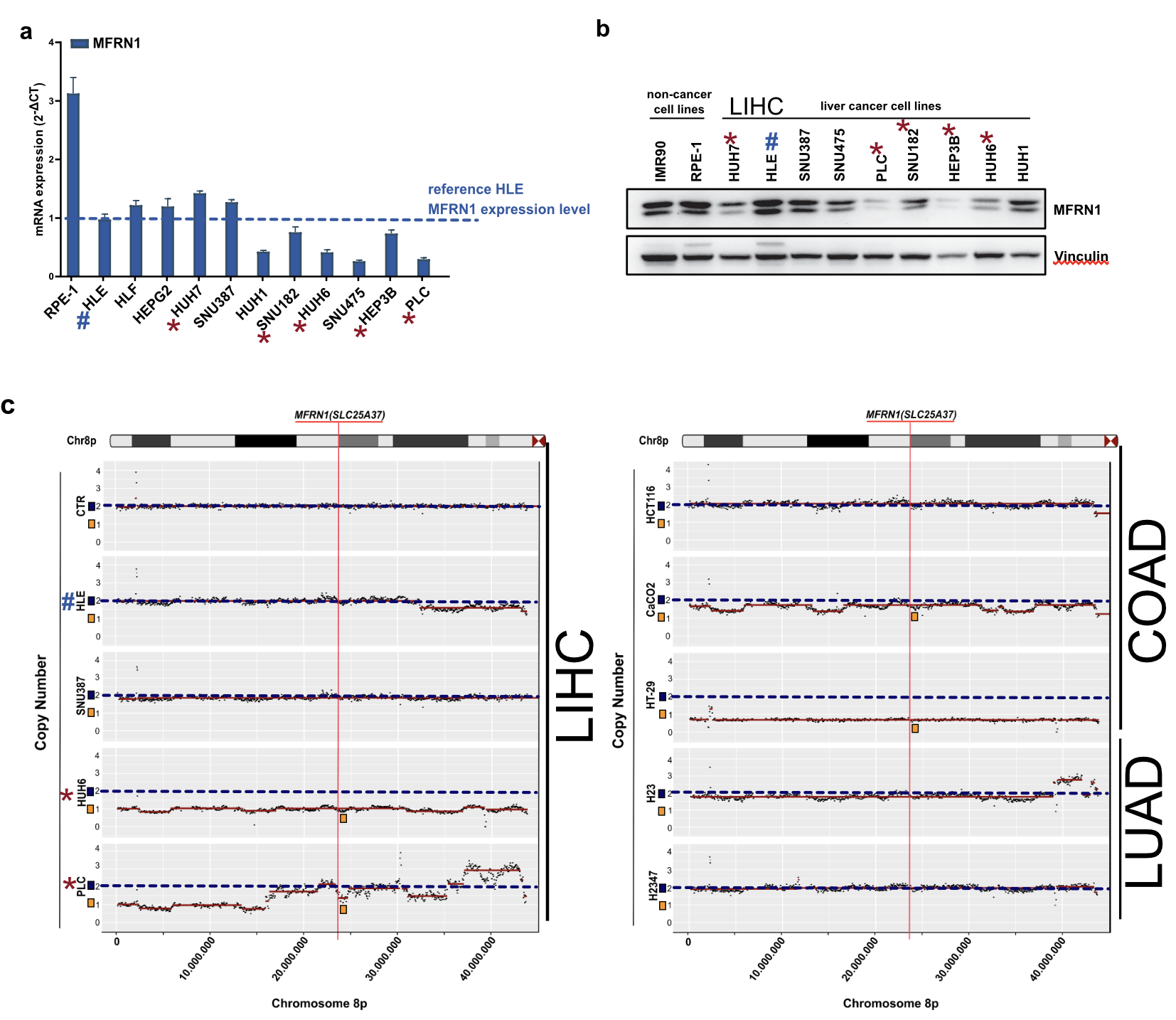


**Fig S2: MFRN1 expression in established cancer cell lines used in this study.**

**a)** Quantitative real-time PCR analysis of MFRN1 gene expression using specific FAM-labeled TaqManR hydrolysis probes and normalizing individual mRNA expression levels to HPRT1. The mean +/- SD of the mRNA level from three individual replicates are shown. Blue line indicates MFRN1 transcript expression observed in HLE cell line nominated as MFRN1-high. Blue hastag indicates HLE cell line **b)** Immunoblot analysis of whole cell lysates from established liver cancer cell lines probed for MFRN1. Vinculin was used as loading control. Representative results are shown. n=3 for MFRN1; n=1 for MFRN2 since the antibody was discontinued. **c)** Whole-genome sequencing analysis to infer somatic copy number alterations. Data for chr8p is depicted for indicated cell lines used in this study. Value of 1 indicates heterozygosity (orange mark), 2 indicates homozygosity, Value of 2 indicates homozygosity (blue dotted line and blue mark) , horizontal red dotted line indicates the respective chromosomal state for each cell line , red vertical line indicates MFRN1 localization within chr8p.

Blue hashtag indicates HLE cell line. Red asterisks indicate cell lines categorized as MFRN1-low. asterisks

**Fig S3: in vitro validation of candidate chr8pSL genes**

**a)** T7 endonuclease I assay of individual sgRNAs targeting MFRN1 and MFRN2 in SNU387 cells. **b)** Immunoblot analysis of whole cell lysates from SNU387 probed for MFRN1. **c)** T7 endonuclease I assay of individual sgRNAs targeting chr8pSL genes (see Fig.1c) in SpCas9 expressing SNU387 cells were transduced with lentiviral vectors expressing the respective sgRNA. Each gene was targeted with 2 independent sgRNAs. M: Marker, C: control; G1: sgRNA-1, G2: sgRNA-2. **d)** Competition assays performed in chr8p deleted HUH6- (upper panel) and chr8p non-deleted SNU387(lower panel) cells using two sgRNAs (violet and orange) to individually target each of the 9 candidate chr8SL. Results are shown as the mean +/- SD of the ratio of GFP+ cells compared to t0 for each assay. n=2 biological replicates.  **e)** Colony formation assays corresponding to the competition assays in both HUH6 (upper panel) and SNU387 (lower). 500 cells were plated in triplicates into 12-well plates and grown for 12 days before fixation and staining with crystal violet solution. Shown are representative triplicates. n=2 biological replicates.

**Fig S4: Paralog Lethality Relationship between MFRN1 and MFRN2 in Chr8p deleted cancer cells** **as revealed by RNAi mediated MFRN2 knockdown**

**a)** Knockdown efficiency of shRNAs targeting MFRN2. Immunoblot analysis of whole cell protein lysates from SNU387 cells transfected with retroviral constitutive expression constructs harboring the indicated shRNAs probed for MFRN2. A shRNA targeting Renilla luciferase was used as control (CTR). Marked in red are the two shRNAs used for the further analysis. Representative results are shown. n=2 biological replicates. **b)** Immunoblot of whole cell lysates from SNU387 cells transfected with the DOX-inducible lentiviral shRNA expression constructs and probed for MFRN2. Vinculin was used as a loading control. Cells were cultured in DMEM supplemented with or without 1 μg/ml DOX 3 days before harvest. Representative results are shown. n=1 biological replicates. **c)** Quantitative realtime PCR analysis of MFRN2 gene expression in SNU387 transduced with lentiviral constructs expressing either a sgRNA targeting MFRN1 (sgMFRN1) or a non-targeting sgRNA (sgCTR) and DOX-inducible constructs expressing either of two shRNA targeting MFRN2 (shMFRN2-1, shMFRN2-2) or Renilla luciferase (shRen) after 6 days. Individual sgRNA and shRNA combinations are indicated with (+). Analysis was performed using specific FAM-labeled TaqManR hydrolysis probes and normalizing individual mRNA expression levels to HPRT1. The mean +/- SD of the relative mRNA level . n=3 biological replicates. **d)** Colony formation assays of SNU387 cells identically targeted as before. 500 Blasticidin and Puromycin selected cells were seeded in 6-well plates in triplicates and cultured in DMEM supplemented with 1 μg/ml Dox. After 12 days, cells were fixated and stained with crystal violet solution. n=2 biological replicates. **e)** Competition assay of SNU387 cells targeted with lentiviral expression constructs as described before. SpCas9 competent cell lines expressing either a non-targeting sgRNA (sgCTR) or a sgRNA targeting MFRN1 (sgMFRN1) were mixed with cells transduced with a DOX-inducible lentiviral construct expressing either a non-targeting shRNA (shRen) or one of two shRNA targeting MFRN2 (shMFRN2- 1, shMFRN2-2) together with GFP in a 30:70 ratio. Representative results of one biological with 3 technical replicates are shown as the mean +/- SD of the ratio of GFP+ cells compared to t0 for each assay. n=2 biological replicates **f), g), h)** corresponding to **c),d),e)** with PLC cells. n=2 biological replicates **i)** Competition assay of PLC cells targeted with lentiviral constructs for ectopic expression of MFRN1. SpCas9 competent cell lines expressing either an empty vector control (EV) or an expression construct for MFRN1 (MFRN1-OE) were mixed with cells transduced with a DOX-inducible lentiviral construct expressing either a non-targeting shRNA (shRen) or a shRNA targeting MFRN2 (shMFRN2) together with GFP in a 30:70 ratio. n=2 biological replicates.

**Fig S5: MFRN2 targeting specifically impairs growth of MFRN1 deficient lung and colon cancer cell lines**

**a)** Immunoblot analysis of MFRN1 expression whole cell lysates from established colon cancer cell lines probed for MFRN1. Vinculin was used as loading control. Representative results are shown. n=2 biological replicates.? **b)** Colony formation assay of colon (HCT-116, CaCO2) cell lines transduced with DOX-inducible lentiviral constructs expressing shRNAs targeting either MFRN2 (shMFRN2) or Renilla luciferase (shRen). 500 cells were seeded in 6 well plates in triplicates, cultured in the respective medium supplemented with 1 μg/ml DOX for up to 10 days before fixation and staining with crystal violet solution. Representative results are shown. n=2 biological replicates. **c)** as in **(a**) with indicated colon cancer cell lines. n=2 biological replicates. (**d)** as in b with indicated lung cancer cell lines. n=2 biological replicates. Red asterisks indicate cell lines categorized as MFRN1 low.

**Fig S6 : MFRN2 targeting impairs mitochondrial function and causes cell death in liver cancer cell lines with low MFRN1 expression**

**a)** Sea horse Mito Stress assay PLC cells were targeted with the indicated sgRNA and shRNA combinations and analyzed for their OCR in a 96 well format before and after the addition of the complex specific inhibitors after 0 days (left) and 6 days (right). Cells were seeded in triplicates one day prior to measurement. n=2 biological replicates. **b)** immunoblot analysis of whole cell lysates from PLC cells treated as indicated and probed for yH2AX. Vinculin was used as a loading control. Representative results are shown. n=2 biological replicates. **c)** Cell cycle analysis of PLC cells transduced with the indicated plasmids after being cultured in DOX containing medium for 6 days. At the indicated time points, cells were harvested ethanol fixated and stained with a 0,1 % (v/v) TritonX-100-solution containing 1 μg/ml DAPI (Ex/Em: 358/461nm). Acquisition was performed using the BD LSR Fortessa flow cytometer (BD, Germany) and the BD FACS Diva software v8.0.1. Analysis generation was performed with FlowJo v10. Graphs show mean + SD for n= 3 biological replicates. **d) I**mmunoblot analysis of whole cell lysates from PLC cells treated as described above and probed for cleaved PARP-1. Vinculin was used as a loading control. Representative results are shown. n=2 biological replicates. **e)** Sea horse Mito Stress assay PLC cells were targeted with the indicated MFRN1 overexpression expression construct- and shRNA combinations and analyzed for their OCR in a 96 well format before and after the addition of the complex specific inhibitors after 6 days. Cells were seeded in triplicates one day prior to measurement. n=2 biological replicates**f)** Immunoblot analysis of whole cell lysates from PLC cells treated as indicated and probed for PARP-1 and cleaved PARP-1. Vinculin was used as a loading control. Representative results are shown. n=2 biological replicates .

**Fig S7: MFRN2 targeting impairs in vivo tumor growth of MFRN1 deficient murine liver cancer cells**

**a)** T7 endonuclease I assay of Mfrn1 and Mfrn2 targeting sgRNAs. SpCas9 expressing cells were transduced with lentiviral vectors encoding the respective sgRNAs. Each gene was targeted with 2 independent sgRNAs. M: Marker, CTR: control; G1: sgRNA-1, G2: sgRNA-2. n=2. **b)** immunoblot analysis of whole cell lysates from primary murine liver cancer cells with Myc/Trp53 background probed for Mfrn1. Vinculin was used as a loading control. Representative results are shown. n=2. **c)** Quantitative real-time PCR analysis of Mfrn2 gene expression in Myc/Trp53 primary murine liver cancer cells transduced with retroviral constructs expressing shRNA targeting Mfrn2 or Renilla luciferase (shRen). Individual shRNAs are indicated by numbers. Analysis was performed using the SYBR-Green detection format and normalizing individual mRNA expression levels to CTR cells harboring a shRNA targeting Renilla luciferase (shRen). The analysis was performed by the ΔΔCT method. Shown is the mean +/- SD of the relative mRNA level. n=3 biological replicates. **d)** Colony formation assays of Myc/Trp53 primary murine liver cancer cells identically targeted as in a. 500 Puromycin selected cells were seeded in 6-well plates in triplicates. After 12 days cells were fixated and staining with crystal violet solution. n=2 biological. **e)** Competition of primary murine liver cancer cell lines with Myc/Trp53- background harboring DOX-inducible lentiviral construct expressing either of two shRNAs targeting Mfrn2 (shMfrn2.1; shMfrn2.2) or Renilla luciferase (shRen) and GFP as well as a lentiviral construct expressing SpCas9 together with either a control sgRNA (sgCTR) or a Mfrn1 targeting sgRNA (sgMfrn1). Cells were cultured in DMEM supplemented with 1 μg/ml DOX and seeded as described in b. Results shown as the mean +/- SD of the ratio of GFP+ cells compared to t0 for each assay with 3 technical replicates. Representative results are shown. N=2 biological replicates. **f)** Colony formation assays corresponding to **e**). N=2 biological replicates**. g)** Primary murine Myc/Trp53 liver cancer cells stably expressing SpCas9 with either sgMfrn1 or sgCTR together with a construct expression either shMfrn2.1, shMfrn2.2 or shRenilla (shRen) were sc. injected into NMRI nude mice (n=5 female mice per group). After reaching a tumor volume of 100 mm3 mice were given a DOX containing diet and tumor size measured periodically.

**Fig S8: MFRN1 and 2 expression analysis across human tissue and tumor entities**

**a)** Expression profile of MFRN1 (left panel) and MFRN2 (right panel) in different tissues**.** Data derived from GTEx^22^ .**b)** Expression profile of MFRN1 (left panel) and MFRN2 (right panel) in different tumor entities**.** Data derived from GEPIA2^21^. **c)** Left: Representative images of TMA patient samples individually scored (MFRN1-Score) according to their staining intensities as indicated below. Right: Donut diagram showing the average MFRN1-Score distribution among the indicated tissue samples next to the individual wedges. Colors represent the respective MFRN1-Score as indicated in the legend. Numbers within each wedge are given as the percentage of the total number of counted samples of different tissue origin. **d)** as in **c)** for ovarian-, prostate-, breast- pancreatic, - colon, -and lung cancer samples.
